# The Hidden Complexities of Electrochemically Active Surface Area Measurements

**DOI:** 10.1021/acsenergylett.5c04204

**Published:** 2026-02-11

**Authors:** Jon Bjarke Valbæk Mygind, Marcel J. Rost, María Escudero-Escribano

**Affiliations:** † Catalan Institute of Nanoscience and Nanotechnology, Campus UAB, Bellaterra, 08193 Barcelona, Spain; ‡ Huygens-Kamerlingh Onnes Laboratory, Leiden University, Niels Bohrweg 2, 2333 CA Leiden, The Netherlands; ¶ Catalan Institution for Research and Advanced Studies (ICREA), Passeig de Lluís Companys, 23, 08010 Barcelona, Spain

## Abstract

Surface-area normalization is essential for quantitative comparison in electrochemistry, yet ambiguity in what area represents hampers interpretation and reproducibility. We distinguish the real surface area, a geometric measure of surface roughness and structure, from the electrochemically active surface area, defined as the condition-dependent subset of surface sites participating in a specific faradaic reaction. We clarify how double-layer capacitance and adsorption-limited charge-transfer reactions probe different regions of the electrode surface and how their interpretation and reference values determine whether the result corresponds to an apparent area, the real surface area, or the electrochemically active surface area. We further show that commonly used reference values vary strongly with electrode structure, electrolyte composition, and measurement protocol. To address this, we introduce a formalism based on domain-specific linear combinations of surface contributions that enables structurally consistent area estimates. Finally, we propose normalizing current by active-site count as a direct and reproducible measure of intrinsic activity.

## Graphical abstract

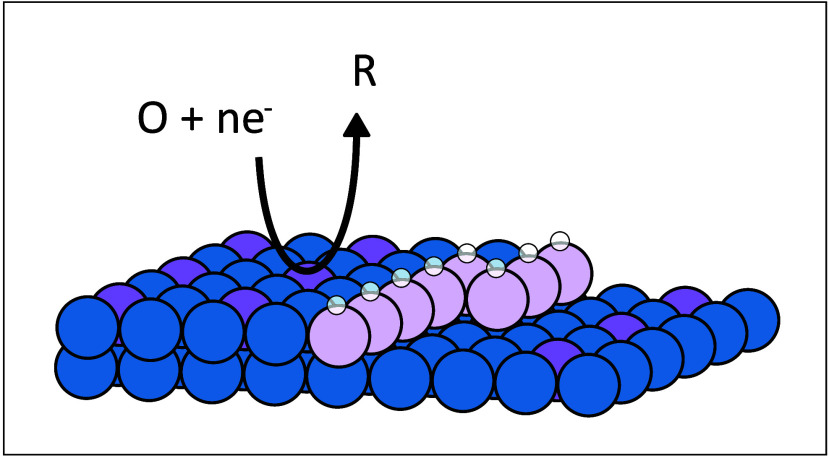

As we strive toward a more sustainable society, the electrification of energy systems has expanded the role of electrochemical science across a wide range of technologies. Understanding the electrode surface area and correctly interpreting electrochemical performance are essential for device optimization and rational electrode design.^1^ In electrocatalysis, it is particularly important to normalize activity metrics by surface area such that they are comparable across studies. This applies to all electrocatalytic reactions, including hydrogen evolution,^2^ oxygen evolution,^3^ CO_2_ reduction,^4^ and methane oxidation.^5^ However, the interpretation of such normalized values depends critically on which surface area is used. The most basic normalization is by the geometric surface area, which refers to the macroscopic, two-dimensional projected area of the electrode as defined by its external dimensions. All electrodes, however, exhibit three-dimensional microscopic roughness that is not captured by the geometric surface area. This additional surface contribution is accounted for in the Real Surface Area (RSA), which denotes the total geometric extent of the electrode, including microscopic irregularities. Normalization by RSA therefore reflects an apparent activity of an electrode, encompassing effects of size, morphology, and roughness. Normalization by the Electrochemically Active Surface Area (ECSA), however, provides a more accurate measure of the intrinsic activity.

ECSA has become a standard metric for current normalization in electrocatalysis, yet it is rarely defined explicitly. Across the literature, ECSA has been treated in three main ways: as equivalent to RSA, as the fraction of the surface participating in electrochemical reactions, and as an operational quantity derived from electrochemical probe measurements (e.g., adsorption-limited faradaic reactions).^3,6–11^ The corresponding measurement methods have been discussed extensively in previous reviews.^10–12^ Several studies have shown that ECSA determinations are highly sensitive to the choice of probe and to details of the experimental protocol.^13–18^ Such discrepancies emphasize that the ECSA is not an intrinsic material constant but an operationally defined quantity that depends on the surface state and measurement conditions. We define the ECSA as the subset of the RSA that participates in a specific faradaic reaction under defined operational conditions, i.e., the population of surface sites active for a specific reaction at a given potential and interfacial environment.

A clear understanding of the electrode surface area is essential for the optimization of electrochemical systems.

In this perspective, we discuss the structure of real electrode surfaces and distinguish between the RSA and ECSA. We address not only how electrochemical methods can be used to probe both quantities, but also highlight the limitations of these approaches. Because such measurements rely on surface-specific reference values, the origin and applicability of these references are crucial in determining what area is actually estimated. We argue that the most accurate area estimates require domain-specific reference values that link electrochemical probe responses to the RSA or ECSA of each surface domain. Finally, we discuss normalization by active-site count as an operational alternative to ECSA-based normalization for assessing intrinsic activity.

## Real Surface Area

RSA refers to the total geometric extent of the electrode surface, including all terraces, steps, defects, overhangs, and grain boundaries—essentially the atomic-scale landscape of the electrode. It is a three-dimensional surface area that differs from its two-dimensional projected geometric area. A convenient dimensionless descriptor of this deviation is the roughness factor

1 $$R_{f}=\frac{A_{\mathrm{RSA}}}{A_{\mathrm{geo}}}$$

where *A*_RSA_ is the true three-dimensional surface area and *A*_geo_ is the geometric surface area, defined as the two-dimensional projection of *A*_RSA_ onto a nominally flat plane. *R*_*f*_ approaches unity for ideally smooth surfaces and increases with increasing roughness. This metric captures how real surfaces deviate from their geometric projection due to microscopic irregularities inherent to practical electrodes.

Real electrode surfaces exhibit a wide range of geometries, from well-defined single-crystal surfaces to polycrystalline and nanoparticle surfaces (Figure 1). Polycrystalline surfaces (Figure 1c) consist of grains separated by grain boundaries, and all electrode types expose crystallographic facets, which are built up from terraces, steps, and kinks.^19^ These facets give rise to diverse adsorption sites, including atop, bridge, and hollow sites on terraces (Figure 1a), as well as kink, edge, and corner sites on under-coordinated regions (Figure 1b and 1d). Even single-crystal surfaces exhibit imperfections such as atomic steps and kinks.^20^ Together, these features determine the real surface area and the diversity of sites that govern electrochemical and electrocatalytic behavior.

**Figure 1:**
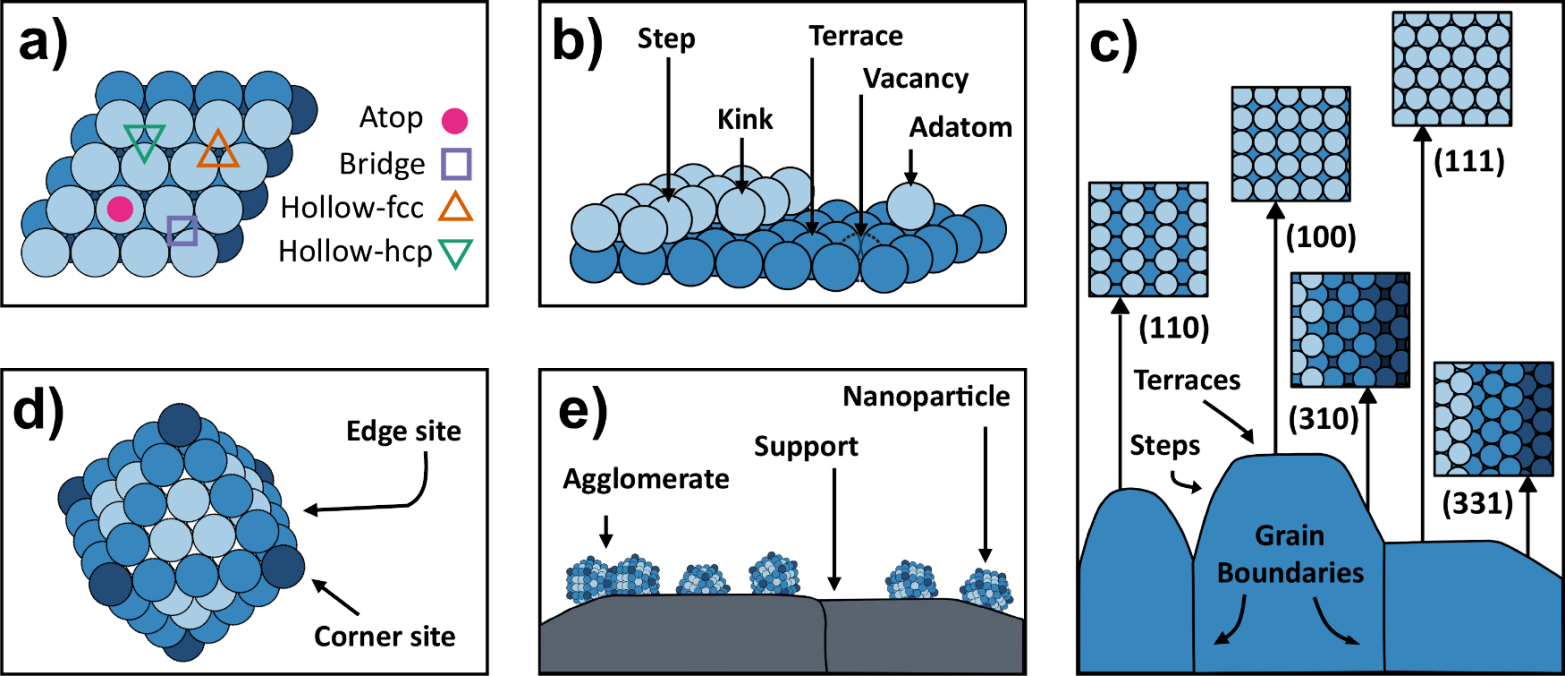
Schematic illustration of electrode surface complexity: (a) top view of a (111) surface indicating different adsorption sites, (b) single-crystal surface showing a terrace, a monatomic step, a kink, an adatom, and a vacancy, (c) cross-sectional view of a polycrystalline surface showing individual grains separated by grain boundaries, including insets of selected low- and high-index facets, (d) octahedral nanoparticle with (111) facets and exposed edge and corner sites indicated, and (e) supported cuboctahedral nanoparticles, including an agglomerate. All structures shown are based on face-centered cubic lattices.

Different preparation methods affect surface structure in distinct ways. Mechanical polishing (e.g., using alumina or diamond slurries) leaves behind grinding particles on the surface and in the polishing grooves. While mechanical polishing can effectively reduce large-scale roughness on uneven surfaces, it also introduces deformation in well-ordered materials, thereby possibly destroying the crystallographic structure.^21^ Less mechanical damage is typically introduced when electropolishing, which relies on controlled anodic dissolution of the electrode surface rather than abrasive contact. When performed under carefully controlled electrochemical conditions, electropolishing can yield highly ordered surfaces.^22,23^ Depending on the applied conditions, electrochemical treatments can also increase surface roughness and alter the distribution of crystallographic microfacets.^24^ Electrochemical oxidation–reduction cycling is a widely used method to condition electrode surfaces, as it leads to reproducible voltammetric features. Such conditioning has been shown to involve electrode dissolution and nanoscale surface roughening, which can improve apparent reproducibility.^25–27^ The exact outcome of electrode preparation is condition dependent, highlighting the need for well-defined protocols.

Annealing generally lowers surface roughness by allowing surface atoms to reorganize and minimize the total free energy of the electrode.^28^ However, depending on the specific conditions, the outcome can be counterintuitive: thermodynamics may favor reconstructions, step bunching, phase transitions, or even three-dimensional roughening.^29–31^ Similar energetic considerations apply at the nanoscale, where annealing can drive major reshaping through Ostwald or Smoluchowski ripening, promoting coarsening or agglomeration that reduce dispersion and modify the accessible surface area.^32,33^ Although the Wulff construction defines equilibrium shapes through surface free-energy minimization,^34^ real nanoparticles often deviate because finite-size, strain, and support effects modify their energetics.^35,36^ This is clearly illustrated by the melting-point depression of gold nanoparticles, which melt hundreds of kelvin below the bulk value.^37^

Since surface preparation can markedly alter the electrode surface, the surface area should be determined after the final preparation steps. The RSA of the resulting electrode surface can be estimated ex situ using a variety of techniques, including scanning probe microscopy,^38–40^ electron microscopy,^10,39,41^ gas adsorption methods,^42,43^ and optical techniques, such as interferometry or ellipsometry.^44,45^ The accuracy of RSA determination depends on both the quality of the measurement and its interpretation, as well as on the suitability of the technique for the surface under study. For instance, scanning probe microscopy provides atomic-scale resolution but becomes challenging for rough surfaces due to tip convolution effects. Gas adsorption, while capable of quantifying large accessible areas, is probe-selective and, for weakly physisorbed probes, requires sufficient total uptake to exceed experimental noise, which in practice often necessitates high accessible surface area. Electron microscopy enables direct imaging but lacks intrinsic height information, so conversion of nanoparticle images into surface area relies on geometric assumptions (often spherical) and sufficiently large sample sets for statistical reliability. These examples emphasize that RSA determination is method-dependent, with each technique’s suitability dictated by the characteristics of the surface under study.

Beyond geometric structure, the local electronic environment of a surface is equally important. Geometric and electronic structure are physically coupled: any structural modification of an electrode necessarily perturbs its electronic structure. Structural variations such as steps, grain boundaries, and strain therefore directly modify the local electronic environment. In general, the resulting surface electronic states are determined by element type and alloying, crystallographic orientation, strain and stress, morphology, finite-size effects (e.g., nanoparticles), grain-boundary network structure and texture, and support interactions.^46–48^ Electronic structure influences the signals obtained from the ex situ characterization techniques discussed above, but with technique-dependent sensitivity. Crucially, it determines the electrochemical response of an electrode surface, defining both the nature of charge-transfer reactions and the capacitive behavior associated with the electrochemical double layer.^49,50^

The electrochemical response of a surface reflects its geometric and electronic structure, shaped by the interfacial environment and the operating conditions.

The RSA can be probed electrochemically by measuring the differential capacitance associated with the double layer. However, the effectiveness of this probing depends on both the electrode surface and the electrolyte, as the local surface potential must be sufficiently screened for all regions of the electrode surface to contribute. In practice, the more porous or rough the surface is, the higher the ionic strength and the longer the charging time scale are required to ensure that the entire RSA contributes to the measured capacitance.^51,52^ Accordingly, differential capacitance measurements obtained by either capacitive cycling (Figure 2a) or electrochemical impedance spectroscopy (Figure 2b) should be interpreted within these constraints.

**Figure 2:**
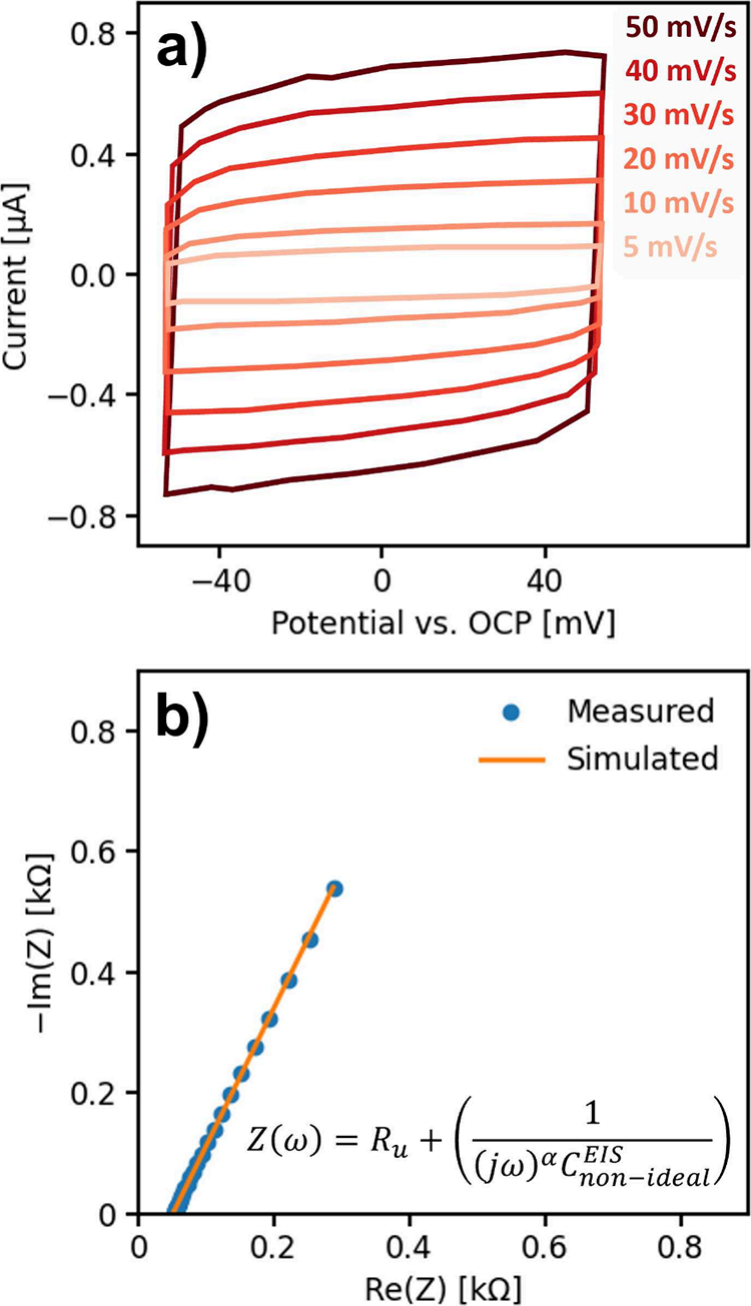
Capacitance determination by (a) cyclic voltammetry in a potential region chosen to minimize faradaic contributions, recorded at varying scan rates, where the capacitive current scales approximately linearly with scan rate, enabling extraction of the double-layer capacitance (see, e.g., ref^53^ for representative linear fits used in practice), and (b) electrochemical impedance spectroscopy, where the Nyquist response is fitted using a constant-phase-element (CPE) model. In (a), the double-layer capacitance is estimated more reliably when the capacitive current shows a stronger linear association with scan rate and faradaic contributions are effectively suppressed, whereas in (b), more accurate estimates come from α values close to unity and the use of a physically reasonable equivalent circuit. Figure redrawn from data in refs 53,54

The differential capacitance can be estimated from capacitive cycling (Figure 2), under the assumption that it remains constant within the chosen double-layer potential window. This analysis further requires a potential window in which faradaic processes and other noncapacitive contributions are negligible, such that the measured current is dominated by charging and discharging of the electrochemical double layer. Under these conditions, the double-layer capacitance *C*_dl_ is obtained from a linear fit of the current *I* as a function of scan rate ν, as shown in eq 2.^53^

2 $$I=C_{\mathrm{dl}}\nu$$

Electrochemical impedance spectroscopy can be used to determine the differential capacitance at a chosen potential, by applying a small sinusoidal perturbation around a chosen potential. This approach requires fitting the data to an equivalent circuit that represents the underlying processes within the frequency range probed. The fewer contributing processes, the easier it is to identify a reliable model. For this reason, the differential capacitance is more easily evaluated by performing the measurement in the double-layer regime, at a potential where the system remains electrochemically stable and over a frequency range where noncapacitive contributions (e.g., faradaic reactions) are negligible. The impedance of an ideal capacitor is given by eq 3

3 $$Z\left(\omega \right)=\frac{1}{j\omega C_{\mathrm{dl}}}$$

here, *j* is the imaginary unit and ω the angular frequency of the applied sinusoidal perturbation. Even under conditions where only the double-layer capacitance is evaluated, modeling the impedance response with an ideal capacitor is often insufficient. This is because, in electrochemical systems, we probe a macroscopic electrode–electrolyte interface that inherently contains microscopic subdomains and heterogeneities. Each microscopic region, characterized by its own capacitance and time constant, would therefore require a distinct circuit element to describe the overall response. The combined effect of these many elements produces nonideal capacitive behavior, which can be modeled by a constant phase element, as described in eq 4.

4 $$Z\left(\omega \right)=\frac{1}{{\left(j\omega \right)}^{\alpha }C_{\mathrm{non}‐\mathrm{ideal}}}$$

The parameter α quantifies the deviation from ideal capacitive behavior, with values closer to 1 indicating more ideal behavior. When α deviates from unity, the fitted capacitance no longer corresponds to one unique physical capacitor but instead represents an effective, phenomenological parameter that reflects a distribution of interfacial time constants. As α decreases, its interpretability as a direct measure of interfacial charge storage reduces. While such values may still serve as operational proxies for comparative studies under carefully controlled conditions, they should be interpreted with caution.^55–58^

The impedance-based method holds considerable promise for broad applicability. For instance, one study extracted the surface area associated with the ECSA of oxide nanoparticles by constructing a comprehensive equivalent circuit, probing a region of specific adsorption, and analyzing the resulting adsorption capacitance.^38^ Such approaches are powerful but also illustrate a challenge: equivalent circuits are simplified representations of real interfacial behavior. Nonideal elements may reproduce experimental data, but uncertainty in their microscopic interpretation propagates into the evaluated differential capacitance and, consequently, into the estimated surface area. This highlights the importance of careful model validation and interpretation.

## Electrochemically Active Surface Area

In electrocatalysis, the ECSA is a key quantity, as it defines the portion of the electrode surface that actually participates in faradaic charge-transfer reactions through direct surface–reactant interactions. However, as illustrated in Figure 1, real electrode surfaces expose a distribution of adsorption sites, each with distinct local coordination, electronic structure, and reactivity. Consequently, the ECSA is not a fixed geometric quantity but depends on the target reaction and on which surface sites are active under the given electrochemical environment. Experimentally, the ECSA is determined from adsorption-limited faradaic reactions or redox transitions of surface species, which give rise to distinct peaks in cyclic voltammograms. The integrated charge associated with these peaks, *Q*_meas_, also contains contributions from double-layer charging, *Q*_dl_, and, depending on the system, also from coadsorbed or coreacting species, *Q*_co_. A background correction is therefore required

5 $$Q_{\mathrm{corr}}=Q_{\mathrm{meas}}-Q_{\mathrm{dl}}-Q_{\mathrm{co}}$$

and only *Q*_corr_ can be related to the ECSA. Figure 3 illustrates representative examples of ECSA determination measurements, including hydrogen underpotential deposition (H-UPD), metal underpotential deposition (M-UPD), CO stripping, and surface oxide reduction.

**Figure 3:**
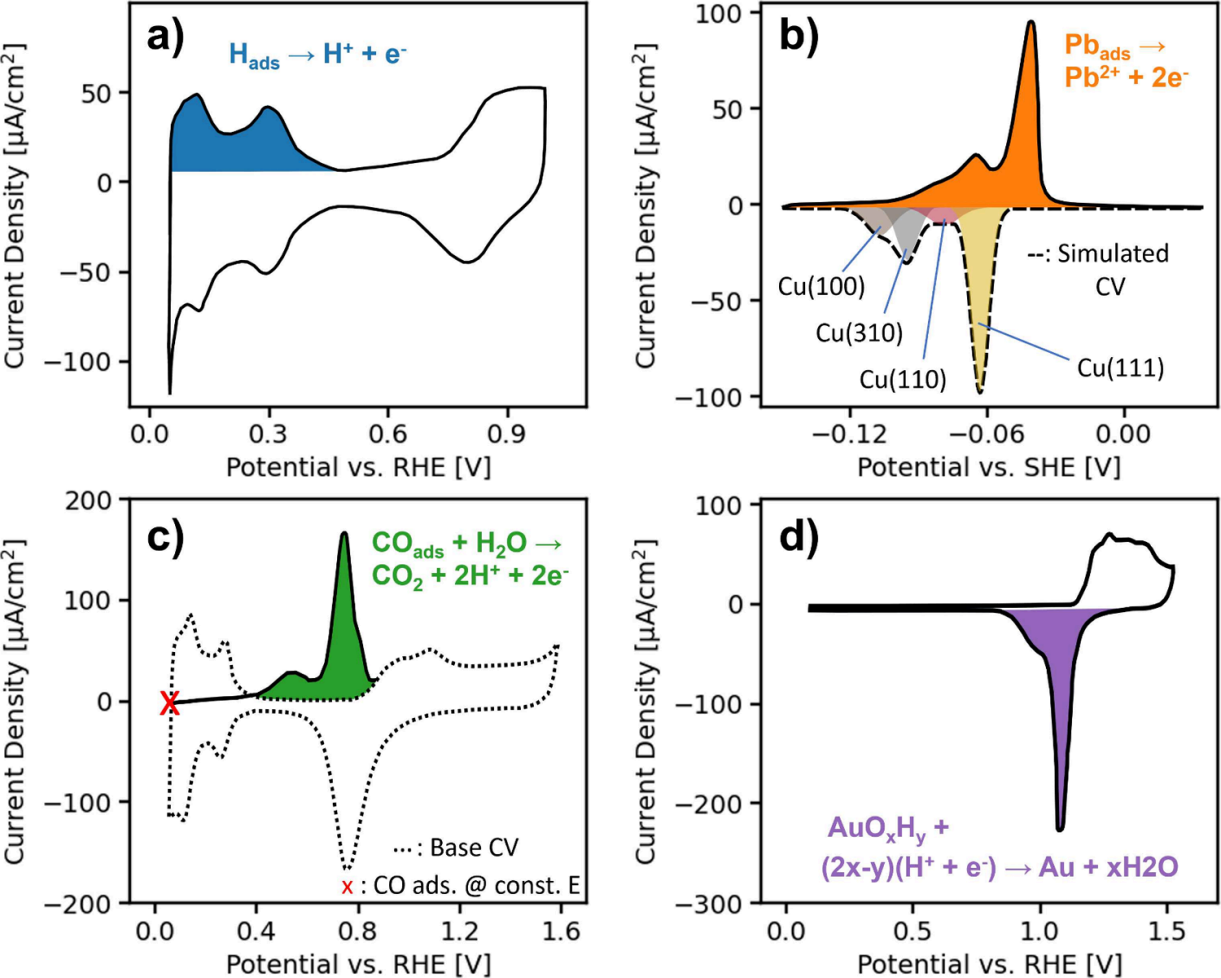
Cyclic voltammograms illustrate four faradaic probe reactions: the shaded areas are used for ECSA estimation. (a) H-UPD on polycrystalline Pt, where the integrated charge corresponds to desorbed hydrogen. (b) M-UPD of Pb on polycrystalline Cu, where distinct deposition peaks reveal crystallographic surface contributions. (c) CO stripping on polycrystalline Pt, where an oxidation peak reflects the charge required to remove a preadsorbed CO layer. (d) Surface oxide reduction on polycrystalline Au, where the integrated reduction peak corresponds to surface oxide removal. The specific appearance of these voltammograms depends on the electrode surface, electrolyte composition, and measurement protocol. Figure redrawn from data in refs 24,59–61

H-UPD (Figure 3a) involves the reversible underpotential deposition (i.e., adsorption) and desorption of hydrogen at potentials more positive than the thermodynamic equilibrium potential for the hydrogen evolution reaction. The total H-UPD charge and its potential profile depend strongly on the electrode surface and the electrolyte environment. For instance, on platinum surfaces, the hydrogen coverage is approximately 0.67 monolayers for (111) terraces and 1.3 for (100) step sites.^62^ These values are inferred from the charge passed during the H-UPD feature and therefore represent the total adsorbed charge in that potential region, which may include contributions beyond pure hydrogen adsorption. It has been argued that the higher apparent coverage along the (100) steps partly arises from concurrent OH adsorption,^63^ which has also been reported on Pt(100) terraces.^64^ Furthermore, depending on the electrolyte composition, coadsorption of ions can contribute additional charge.^65,66^ Consequently, accurately estimating the ECSA from cyclic voltammetry requires careful accounting for both the true double-layer contribution and coadsorption effects.

Metal underpotential deposition (M-UPD) involves the underpotential adsorption and reduction of metal ions at potentials more positive than their Nernst equilibrium potential. Like H-UPD, the other faradaic probe reactions are also subject to artifacts that limit interpretation. In M-UPD, anion coadsorption can alter the measured charge, while the deposition process itself may rearrange surface-near atoms.^67,68^ In CO stripping, CO is first adsorbed at a constant potential, after which the electrolyte is purged to remove dissolved CO: the adsorbed layer is then oxidatively stripped during a potential sweep. Here, concurrent OH coadsorption can increase the integrated charge, and the CO adsorption–oxidation sequence can alter alloy surface composition by preferential oxidation or segregation.^69,70^ Finally, surface-oxide reduction probes the ECSA via the cathodic charge associated with reducing an electrochemically formed oxide, the oxidation–reduction sequence itself can induce restructuring and roughening of the surface.^26,71–73^ These examples are not exhaustive but illustrate that faradaic probe methods may reflect processes other than the intended reaction and may also modify the electrode surface, and thus do not necessarily represent the active sites of interest under operational electrocatalytic conditions.

In electrocatalysis, the ECSA is a key quantity for assessing intrinsic activity, as it represents the surface sites active for a specific reaction under operational conditions. Because ECSA values derived from probe reactions reflect only the sites accessible to that probe, they may differ substantially from those relevant to the target electrocatalytic reaction. One should therefore carefully consider how well the chosen probe reaction mimics the active sites of interest. For instance, in the case of Cu electrodes for CO_2_ reduction, CO-displacement is likely to provide a more accurate representation of active sites than alternative methods.^74^ For the oxygen reduction reaction on Fe–N–C catalysts, it has been shown that ${{\mathrm{NO}}_{2}}^{-}$ adsorption serves as an effective probe of the catalytically active sites.^75^ Ultimately, the ECSA is not an intrinsic property of the electrode but a condition-dependent quantity that reflects the subset of surface sites participating in a specific electrocatalytic reaction.

The electrochemically active surface area can only be defined for a specific reaction occurring at the atomistic configuration present at the moment of the reaction.

Figure 4 depicts a conceptual understanding of surface areas and their electrochemical probing: (a) the RSA, representing the three-dimensional surface across all microstructural features; (b) the portion of the surface probed via the electrochemical double layer; (c) the ECSA associated with an adsorption-limited faradaic reaction (e.g., H-UPD); and (d) the ECSA associated with a kinetically or mass-transport-limited electrocatalytic reaction. The schematic in (b) is simplified, as it omits specific adsorption and the molecular arrangement of electrolyte species, both of which depend on the details of the capacitance measurement. Moreover, the extent of the areas shown in (c) and (d) depends on the reaction under consideration, since different electrocatalytic processes engage distinct subsets of surface sites.

**Figure 4:**
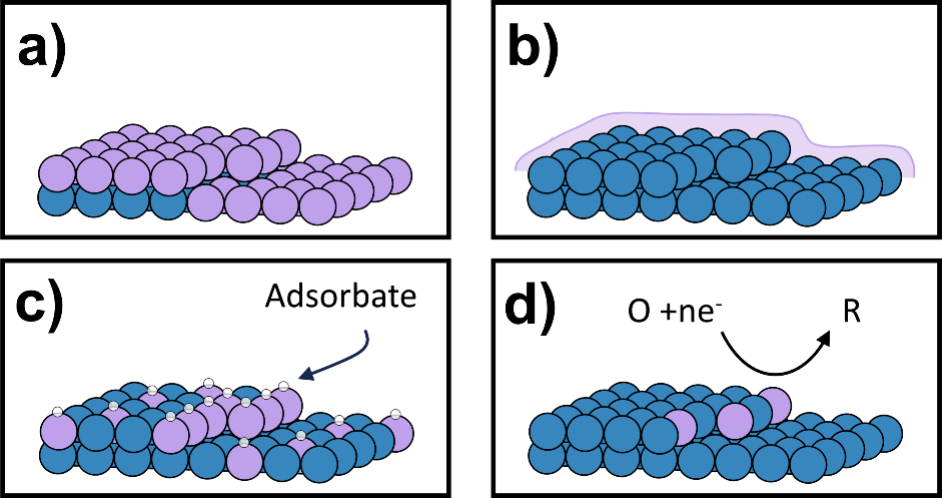
Conceptual definitions of surface areas and their electrochemical probing: (a) real surface area; (b) surface area probed via the differential capacitance of the electrochemical double layer; (c) electrochemically active surface area associated with an adsorption-limited faradaic reaction, where white spheres denote the adsorbate; and (d) electrochemically active surface area associated with a kinetically or mass-transport-limited faradaic reaction. Blue spheres represent the electrode lattice, while purple spheres and the shaded contour in panel (b) indicate regions contributing to each defined surface area.

## Operational and Quantitative Determination of Surface Area

Up to this point, we have discussed how different electrochemical methods can be used to probe various surface areas. To convert such measurements into a quantitative surface area, a reference value per unit area is required. The surface area of the sample electrode can then be evaluated from the measured capacitance or integrated charge using eqs 6a and 6b

6a $$A^{\left(sample\right)}=\frac{C^{\left(sample\right)}}{C_{s}}$$

6b $$A^{\left(sample\right)}=\frac{Q^{\left(sample\right)}}{Q_{s}}$$

where *C* ^(sample)^ and *Q* ^(sample)^ denote the double-layer capacitance and the adsorption-limited faradaic charge associated with the sample electrode, and *C*_s_ and *Q*_s_ are the corresponding surface-specific reference values. A persistent difficulty in determining the surface area of electrodes is the establishment of reliable reference values.

Historically, for H-UPD on polycrystalline and nanoparticle platinum surfaces, a value of 210 μC cm^–2^ has been widely accepted.^11,12^ Frumkin reported this value in the 1960s, referring to unpublished low-temperature krypton adsorption measurements on platinized platinum.^76^ Brummer rationalized it to the assumption of a full hydrogen coverage on Pt(100), with a site density of 13.03 atoms nm^–2^.^77^ In the 1990s, however, Trasatti explicitly noted that *“The absolute significance of the accepted Q*_*H*,*s*_ *is questionable.”* ^12^ This uncertainty arises because the H-UPD charge depends strongly on surface structure and electrolyte composition, meaning that a single reference value such as 210 μC cm^–2^ cannot universally represent either polycrystalline or nanoparticulate platinum surfaces.

For CO stripping on polycrystalline platinum, a reference value of 420 μC cm^–2^ has often been applied.^11^ Similarly, for surface-oxide reduction, reference values of 420 μC cm^–2^ for platinum, 390 μC cm^–2^ for gold, and 516 μC cm^–2^ for nickel are commonly used.^12,78^ All these values are rationalized by assuming full monolayer coverage on the corresponding metal (100) terrace, with two electrons transferred per terrace atom. However, the measured charge and the inferred coverage depend strongly on experimental conditions, including electrode structure and electrolyte composition.

Another common approach is the application of a smooth surface (i.e., *R*_*f*_ = 1) as a normalization reference, measuring either the charge of an adsorption-limited faradaic reaction or the double-layer capacitance. The corresponding reference quantities $C_{smooth}^{\left(ref\right)}$ and $Q_{smooth}^{\left(ref\right)}$ are determined for a surface of known RSA, $A_{RSA,smooth}^{\left(ref\right)}$ . When these values are applied to a different sample, the resulting area represents only a first-order approximation of the RSA, assuming linear proportionality between the measured quantity and surface area. Unless the electrode surface of the sample is identical to that of the smooth reference, higher-order effects (e.g., electrode surface structure) introduce deviations, and the calculated value corresponds to an apparent rather than a physically defined area, as expressed in eqs 7a and 7b.

7a $$A_{apparent}^{\left(sample\right)}=\frac{C^{\left(sample\right)}A_{RSA,smooth}^{\left(ref\right)}}{C_{smooth}^{\left(ref\right)}}$$

7b $$A_{apparent}^{\left(sample\right)}=\frac{Q^{\left(sample\right)}A_{RSA,smooth}^{\left(ref\right)}}{Q_{smooth}^{\left(ref\right)}}$$

For example, consider using a smooth polycrystalline Ir surface (i.e., *R*_*f*_ = 1) as a reference, where the two-dimensional geometric area equals $A_{RSA,smooth}^{\left(ref\right)}$ . CO stripping is performed on this surface to determine $Q_{smooth}^{\left(ref\right)}$ . The resulting reference value is then applied to estimate the surface area of a highly porous, electrodeposited Ir electrode using the same method. However, the smooth and the electrodeposited Ir surfaces are likely to exhibit different distributions of crystallographic orientations. Moreover, CO coverage on (111), (100), and (110) terraces has been shown to vary significantly.^69^ Consequently, the apparent area obtained for the porous electrode remains referenced to $A_{RSA,smooth}^{\left(ref\right)}$ but deviates from it. The magnitude of this deviation reflects the structural disparity between the two electrode surfaces, assuming identical measurement conditions such as electrolyte composition and procedure.

## Reference Variability

Estimating electrode surface areas using electrochemical methods requires a reference value. However, both the charge associated with an adsorption-limited faradaic probe and the measured double-layer capacitance depend sensitively on the specific measurement conditions. Figures 5 and 6 illustrate the resulting variability in reference values, all of which are anchored to the RSA.

**Figure 5:**
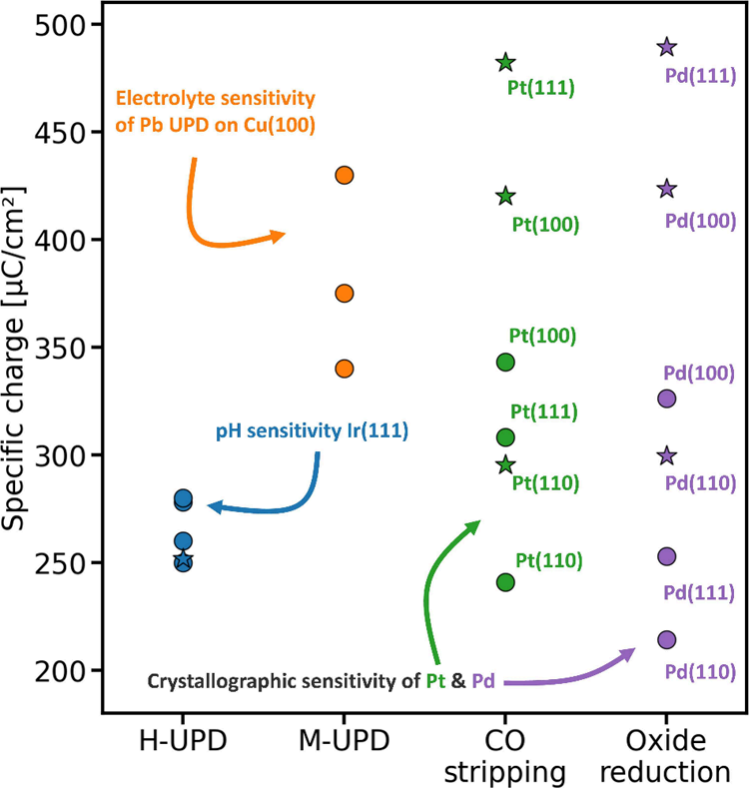
Reference normalization charges: Circles represent experimentally determined values after background subtraction: blue for H-UPD,^79^ orange for M-UPD,^24,80,81^ green for CO stripping^69^ and purple for oxide reduction.^82^ Stars indicate theoretical estimates corresponding to full-monolayer coverage using stoichiometric expectations for the number of electrons transferred and atomic packing densities corresponding to the crystallographic orientation. Experimental values shown are representative literature reports rather than averages over independently prepared electrodes; error bars are therefore not shown.

**Figure 6:**
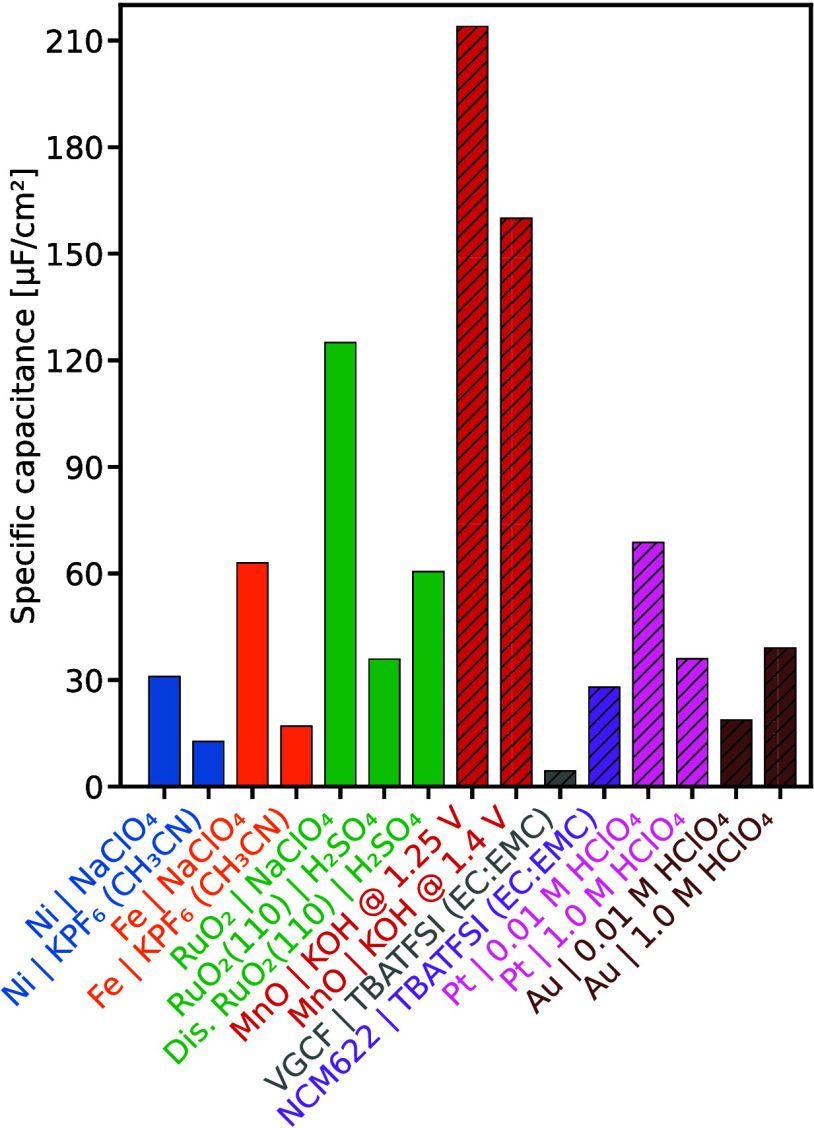
Reported normalization capacitance values for various electrode–electrolyte systems, determined by cyclic voltammetry (solid bars) and electrochemical impedance spectroscopy (hatched bars). Unless otherwise indicated, aqueous electrolytes were used; organic solvents are shown in parentheses. VGCF denotes vapor-grown carbon fibers, and NCM622 denotes ${Li}_{1.01}{\left({Ni}_{0.6}{Co}_{0.2}{Mn}_{0.2}\right)}_{0.99}O_{2}$ . Data compiled from refs 54,61,87–89 Values are representative literature reports rather than averages over independently prepared electrodes; error bars are therefore not shown.

Figure 5 shows the surface-specific charge values normalized to the RSA. These values are either determined experimentally using a smooth surface (i.e., *R*_*f*_ = 1), or theoretically calculated by assuming full-monolayer coverage, expected electron transfer per surface atom, and the corresponding atomic packing densities. Except for H-UPD on Ir(111), the experimentally determined values are consistently lower than the theoretical estimates, indicating submonolayer coverages. The experimentally determined values for each probe reaction also vary noticeably among themselves, reflecting the influence of the electrode’s crystallographic orientation and the electrolyte composition used during the measurement. These variations arise because both adsorption coverage and surface-site density are orientation-dependent, while the electrolyte composition further influences the coverages of adsorbed species.^67,69,83^ Any modification of the local electronic structure, whether arising from orientation, elemental identity, alloying, or strain, can significantly influence the response of a probe reaction. For instance, when comparing pure Pt surfaces to alloyed Pt electrodes, both CO adsorption and hydrogen adsorption are markedly altered, reflecting contributions from ligand effects and strain effects.^48,84,85^ Furthermore, the measured surface-specific charge of the probe reaction is sensitive to experimental parameters such as scan rate and potential limits. For instance, M-UPD exhibits diffusion-related scan-rate dependence, while oxidation–reduction cycling can induce surface restructuring depending on the applied scan rate and potential window.^14,86^ These examples illustrate that charges extracted from adsorption-limited faradaic reactions depend strongly on the specific physical and electrochemical conditions.

This condition-dependent variability has direct consequences for how surface-area-normalized activities are interpreted and compared across studies, as illustrated in Figure 5, including in widely studied systems such as Pt-based fuel-cell catalysis. Commonly used reference values (e.g., 210 μC cm^–2^ for H-UPD or 420 μC cm^–2^ for CO stripping on polycrystalline Pt) implicitly assume surface structures and adsorption configurations that are rarely representative of real polycrystalline, alloyed, or nanostructured electrodes. As illustrated in Figure 5, the surface-specific charge associated with CO stripping consistently deviates from 420 μC cm^–2^ and varies significantly with crystallographic orientation. Applying a single fixed reference value across structurally dissimilar surfaces can therefore shift apparent activity trends without any corresponding change in the underlying catalytic behavior. Such normalization-induced variability does not invalidate surface-area normalization per se, but it does introduce an additional, often unquantified source of uncertainty that must be considered when comparing activity, stability, or performance metrics between different electrodes or catalyst architectures.

Figure 6 shows reported double-layer capacitance values normalized to the RSA. Differences across electrode materials, electrolyte compositions, and measurement procedures highlight the sensitivity to both material properties and experimental conditions. Even nominally identical surfaces can exhibit substantial variations as a function of potential or in the presence of structural disorder.^54,61,88,89^ The extracted capacitance also depends on the measurement configuration and data treatment, including the chosen potential window, scan rate, frequency range, AC amplitude, and equivalent-circuit model.^56^ Overall, the reported values represent a convolution of the true double-layer capacitance with the specific measurement protocol, underscoring the need for well-defined reference conditions.

When capacitance or charge measured on a smooth reference electrode is used to normalize a sample electrode, the experimental conditions, including electrolyte composition and measurement protocol, should be matched as closely as possible, as this reduces procedure-dependent uncertainties in the resulting surface area estimate. However, reproducing the electrode surface itself is not possible, since its characterization is the very objective of the experiment. Consequently, uncertainty associated with the electrode structure is unavoidable when a single smooth electrode is used as the reference.

## Real Surface Area Determination from Linear Combinations

Because a nonsmooth sample electrode is likely to exhibit structural disparity relative to a single smooth reference, an alternative approach is required. One approach is to describe the sample surface as a weighted combination of its surface domains. Polycrystalline electrodes and nanoparticles both exhibit a distribution of crystallographic orientations. These can be quantified using surface X-ray diffraction or electron backscatter diffraction for polycrystalline materials,^90^ and through a combination of electron microscopy and atomistic modeling for nanoparticles.^36^ Domain-specific double layer capacitances, $C_{i}^{\left(ref\right)}$ , or adsorption-limited faradaic charges of a probe reaction, $Q_{i}^{\left(ref\right)}$ , can be obtained on representative single-crystal electrodes, where *i* denotes the domain type. Using these domain-specific reference values, the RSA of the sample can be determined using eqs 8a and 8b (see SI for derivation)

8a $$A_{RSA}^{\left(sample\right)}=\frac{C^{\left(sample\right)}}{\underset{i}{\sum }x_{RSA,i}\frac{C_{i}^{\left(ref\right)}}{A_{RSA,i}^{\left(ref\right)}}}$$

8b $$A_{RSA}^{\left(sample\right)}=\frac{Q^{\left(sample\right)}}{\underset{i}{\sum }x_{RSA,i}\frac{Q_{i}^{\left(ref\right)}}{A_{RSA,i}^{\left(ref\right)}}}$$

where *x*_RSA,*i*_ is the fraction of the sample electrode’s RSA associated with domain type *i* (with *∑*_*i*_ *x*_RSA,*i*_ = 1).

An advantage of adsorption-limited faradaic probe reactions is that their voltammetric features can, in certain cases, be decomposed into domain-specific contributions. This approach has been successfully applied to the deconvolution of crystallographic orientations of polycrystalline and nanoparticle electrodes, for example with H-UPD on platinum and Pb-UPD on copper.^24,62,80,91^ In such cases, the RSA of the sample electrode is determined using the domain-specific charge fractions *x*_Charge,*i*_, as described in eq 9 (see SI for derivation):

9 $$A_{RSA}^{\left(sample\right)}=\underset{i}{\sum }x_{Charge,i}Q^{\left(sample\right)}\left(\frac{A_{RSA,i}^{\left(ref\right)}}{Q_{i}^{\left(ref\right)}}\right)$$

Depending on the electrode system, the accuracy of the RSA obtained from linear combinations depends on how completely the surface structure is characterized and represented. Crystallographic domains defined for single crystals do not map directly onto nanoparticles, as lattice strain and finite-size effects modify surface energetics. Nanoparticles also introduce additional types of surface sites, such as edges and corners, that further diversify the local electronic environment. More generally, any factor that alters the electronic structure, including strain, stress, support interactions, or alloying, can cause deviations from the reference values. In such cases, the estimate does not reflect the true RSA but an apparent RSA whose accuracy is limited by the mismatch between the sample’s surface structure and the structural basis used in the linear combination.

However, even an accurate RSA is not the sole quantity of interest in electrocatalysis. When the aim is to assess intrinsic catalytic activity, attention naturally shifts to the ECSA, defined as the subset of surface sites that participate in a target reaction under operating conditions. Determining the ECSA requires an adsorption-limited faradaic probe reaction that selectively reports on the active sites of the target reaction. Moreover, the area evaluated in the linear combination has to reflect the ECSA rather than the RSA.

## Electrochemically Active Surface Area from Linear Combinations

To determine the ECSA associated with a surface domain *i*, the charge measured from an adsorption-limited probe reaction is compared to the charge expected for full monolayer coverage on that domain. This requires constructing representative periodic surface units that capture the characteristic adsorption environments of domain *i*. Such constructions have been carried out, for example, for Pt(111) and its vicinal surfaces.^62,92,93^ For a given domain, the total charge per unit area corresponding to monolayer coverage is given by eq 10

$$\frac{Q_{unit,i}}{A_{unit,i}}=\frac{N_{sites,i}n_{probe}e}{A_{unit,i}}$$ 10

where *N*_sites,*i*_ is the number of probe-relevant adsorption sites within one unit defined by the area *A*_unit,*i*_, *n*_probe_ is the number of electrons transferred per adsorbed species, and e is the elementary charge. A construction of surface units for Cu(111), Cu(100), Cu(110), and Cu(310) is provided in the Supporting Information, together with an example illustrating the determination of both RSA and ECSA. If the domain-specific area distribution of the sample is known, eq 11 provides the ECSA when combined with reference data obtained from representative single-crystal electrodes.

11 $$A_{ECSA}^{\left(sample\right)}=\underset{i}{\sum }Q_{i}^{\left(ref\right)}\frac{A_{RSA,i}^{\left(sample\right)}}{A_{RSA,i}^{\left(ref\right)}}\left(\frac{A_{unit,i}}{Q_{unit,i}}\right)$$

However, similar to RSA, this is limited by how well the electronic structure of individual surface domains on the sample electrode matches that of the corresponding single-crystal references. If the domain-specific charge distribution is known, the ECSA can be determined by eq 12 without requiring measurements on representative single-crystal reference surfaces.

12 $$A_{ECSA}^{\left(sample\right)}=\underset{i}{\sum }x_{Charge,i}Q^{\left(sample\right)}\left(\frac{A_{unit,i}}{Q_{unit,i}}\right)$$

These expressions provide a formal route for determining the ECSA of a real electrode. Their accuracy depends on the reliable subtraction of nonprobe-related contributions, including double-layer charging and coadsorption effects (see eq 5), as well as any uncertainty in the assumed electron stoichiometry of the probe reaction, *n*_probe_. When ECSA is used to normalize the current associated with a continuous electrocatalytic reaction, the probe reaction must report on sites that are active under the relevant operating conditions. In formulations based on geometric area fractions, this further requires mapping domain-specific responses to single-crystal reference values, an assumption that breaks down when the electronic structure of the surface domains on the sample electrode does not match that of the corresponding single-crystal reference, for example in nanoparticles.

ECSA analysis via unit-charge construction provides more than a normalization factor. By comparing the unit charge associated with domain *i* to the measured charge contribution of the corresponding domain on the sample electrode, information about the relative activity of different surface domains can be obtained, enabling structure–activity relationships for the probe reaction. At the same time, determining the domain-specific unit charge requires identifying the appropriate periodic surface unit and enumerating the adsorption sites within that unit. This task becomes increasingly difficult as the surface grows more complex and may ultimately become impossible to carry out rigorously for highly complex systems. Given this level of complexity and the number of underlying assumptions, a simpler alternative that requires fewer steps and fewer structural inputs can be used for routine applications and for poorly characterized or structurally complex electrocatalysts.

## Active Site Counting

The ECSA represents the contribution of electrochemically active sites expressed in units of area rather than as a direct site count. While ECSA analysis, particularly when combined with structure-resolved unit-charge constructions, can provide detailed information about site identity and activity, such information is not required for routine normalization. An alternative, operational approach is to treat the electrode surface as a black box and infer the number of active sites directly from an adsorption-limited faradaic probe reaction. In this framework, each reaction event corresponds to one active site, where a site is understood as a local interfacial environment defined by the probe reaction. The number of sites sampled by a probe reaction is given by eq 13:

13 $$N_{probe,active}^{\left(sample\right)}=\frac{Q^{\left(sample\right)}}{e\cdot n_{\mathrm{probe}}}$$

The corresponding site-normalized current is then obtained by dividing the target-reaction current by the number of active sites, as given in eq 14, with both probe and target reactions measured on the same electrode.

14 $$I_{target,site}^{\left(sample\right)}=\frac{I_{target}^{\left(sample\right)}}{N_{probe,active}^{\left(sample\right)}}$$

When the target reaction is a continuous electrocatalytic process such that the system performs ongoing turnovers and the rate is limited by surface kinetics, it is appropriate to express the site-normalized current as a turnover frequency (TOF)

15 $$\mathrm{TOF}=\frac{I_{target,site}^{\left(sample\right)}}{n_{\mathrm{target}}\cdot e}$$

where *n*_target_ is the electron stoichiometry of the target electrocatalytic reaction. Normalization by the number of active sites is, in principle, the most direct way to report catalytic activity, provided that the site count reflects the surface state relevant under operating conditions. Similar to ECSA-based normalization, the approach in eq 13 requires that the chosen faradaic probe reaction samples the same population of sites that participate in the target reaction under the applied conditions. However, the surface itself may restructure during operation, exposing or deactivating sites over time.^94–96^

A further limitation arises when the sites sampled by the probe reaction do not correspond to those that govern the rate of the target electrocatalytic reaction. Faradaic probe reactions primarily report on adsorption and desorption processes under their specific conditions, such that sites with stronger binding often contribute disproportionately to the measured probe charge. In contrast, catalytic activity is frequently maximized at sites that bind key reaction intermediates neither too strongly nor too weakly, consistent with the Sabatier principle. As a result, sites that dominate the probe response are not necessarily those that control catalytic turnover, even when both measurements are performed on the same electrode surface.

The accuracy of the extracted site count also depends on removal of nonprobe related charge contributions (i.e., how well *Q*_dl_ and *Q*_co_ capture nonprobe contributions; see eq 5) and any uncertainty in the assumed electron stoichiometry of the probe reaction, *n*_probe_. When a suitable probe reaction is available, site-based normalization provides an operational descriptor of catalytic activity that avoids explicit assumptions about surface geometry or domain structure. However, similar to RSA- and ECSA-based approaches, it does not provide direct information about which specific sites govern the electrocatalytic reaction under operating conditions.

## Research-Driven Comparison

To compare electrochemical responses across different electrodes, the measured current must be normalized. The appropriate normalization depends on the purpose of the analysis. Figure 7 illustrates a simplified, routine-use workflow that highlights the initial considerations guiding this choice.

**Figure 7:**
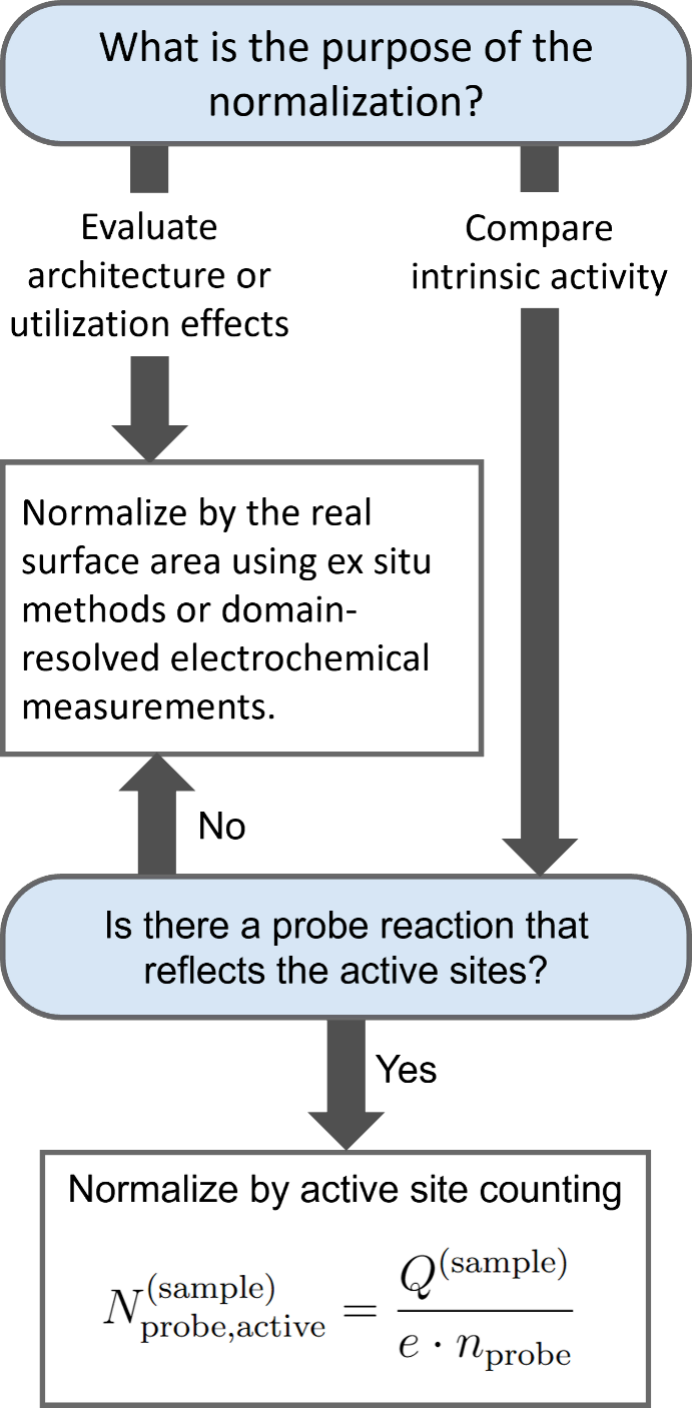
Flow diagram supporting the selection of normalization strategies, shown here as a minimal, routine-use framework.

If the aim is to evaluate how electrode architecture or porosity influences the measured electrochemical response, normalization to the RSA is relevant. If the goal is to compare intrinsic activity between different catalysts, normalization should be evaluated on the basis of the number of reaction-relevant active sites. When such sites cannot be selectively identified using a suitable probe reaction, RSA-based normalization provides a transparent and informative alternative, provided its assumptions are explicitly stated.

RSA, defined here as an absolute geometric quantity of the electrode, contains the most fundamental information about the surface and is therefore always informative. RSA reflects the total geometric extent of the electrode, independent of electrochemical accessibility or reactivity, and serves as a natural complement to ECSA- or active-site-based analyses.

In practice, RSA can be estimated using ex situ characterization techniques, with the resulting value carrying uncertainty relative to the true absolute surface area depending on the details of the procedure. Direct determination of RSA under electrochemical operating conditions is generally not feasible, except in cases where in situ surface-sensitive techniques are available. When RSA is inferred from electrochemical measurements, and where possible, a structurally consistent domain-resolved linear-combination approach should be employed, as it reflects the actual distribution of surface domains within the sample. When a single smooth reference surface is used instead, the resulting RSA should be explicitly reported as an apparent RSA, thereby making the underlying assumptions transparent. Regardless of the method, RSA estimates should be accompanied by a clear discussion of their uncertainty, quantitative where possible and qualitative otherwise.

ECSA, in contrast, is a conditional geometric quantity defined with respect to a specific electrochemical reaction under defined operational conditions. Beyond serving as a normalization factor, ECSA analysis can itself provide valuable insight into electrode characteristics. In particular, combined RSA and ECSA analyses using domain-resolved linear-combination approaches can provide a comprehensive, structure-informed characterization of the electrode surface, while also offering information on domain-specific probe reactivity.

ECSA is not included explicitly as a standalone quantity in the routine-use workflow diagram, not because it is less informative, but because its rigorous determination typically requires additional structural assumptions and model inputs, which increase complexity and uncertainty for routine application, particularly on complex or evolving surfaces. As such, ECSA analysis represents a more complete, structure-informed characterization of the electrode surface, but one whose interpretive power depends critically on the validity of the underlying assumptions.

Active-site counting provides the most direct route to intrinsic activity normalization but yields comparatively limited structural information about the electrode surface. When a probe reaction can be convincingly shown to selectively infer the population of reaction-relevant active sites, active-site-based normalization is well suited for routine comparisons of intrinsic activity.

Comparisons gain meaning only when the normalizations behind them are consistent.

Regardless of whether normalization is based on RSA, ECSA, or active-site counting, the associated assumptions, limitations, and uncertainties must be explicitly stated. In practice, subjecting an electrode to multiple, complementary surface-area characterization techniques can increase the information content available, thereby improving the ability to assess assumptions and limitations. Electrochemical probe reactions represent one important class of such techniques; when they are used, the analysis should address how accurately they reflect the active sites under operational conditions. Because electrode surfaces may restructure, evolve, or change accessibility under applied conditions, such analyses are most informative when performed both before and after operation, unless the relevant surface properties can be assessed in situ or operando. Ultimately, cross-study comparisons are only as meaningful as the transparency and consistency of the normalization on which they are based.

## Outlook

In the broader context of electrochemical science, progress depends not only on the development and optimization of materials and interfaces, but also on the reliability and comparability of how their properties are measured and reported. A clear distinction between the real surface area, the electrochemically active surface area, and the number of active sites is essential for meaningful comparison. The electrochemically active surface area varies with the reaction used to probe the electrode and with the measurement conditions, and thus should not be regarded as a material-specific quantity. Because accurate determination of these quantities is inherently challenging, their uncertainties should be stated explicitly, quantitatively where error sources can be evaluated and qualitatively where they cannot. At the same time, there is a continued need to develop experimental or theoretical strategies that more directly and robustly access the real surface area, the electrochemically active surface area, and the number of active sites under realistic operating conditions. Such approaches are particularly important for capturing dynamic surface and interfacial restructuring, as well as reaction-induced changes in site accessibility and identity. From an experimental perspective, the application of surface-sensitive in situ techniques under reaction conditions could prove particularly valuable, including vibrational spectroscopy, scanning probe microscopy, and surface X-ray methods. Continued methodological development, together with transparent reporting of assumptions and uncertainties, will strengthen the foundations of electrochemical science and accelerate progress in energy technologies.

## Supplementary Material



## Notes

Surface-unit constructions for Cu(111), Cu(100), Cu(110), and Cu(310); derivations of RSA and ECSA expressions; domain-resolved charge analysis; active-site counting methodology; and supporting figures (PDF)

## References

[1] Seh Z. W., Kibsgaard J., Dickens C. F., Chorkendorff I., Nørskov J. K., Jaramillo T. F. (2017). Combining theory and experiment in
electrocatalysis: Insights into materials design. Science.

[2] Hansen J. N., Prats H., Toudahl K. K., Mørch Secher N., Chan K., Kibsgaard J., Chorkendorff I. (2021). Is There Anything
Better than Pt for HER?. ACS Energy Letters.

[3] Wei C., Rao R. R., Peng J., Huang B., Stephens I. E. L., Risch M., Xu Z. J., Shao-Horn Y. (2019). Recommended
Practices and Benchmark Activity for Hydrogen and Oxygen Electrocatalysis
in Water Splitting and Fuel Cells. Adv. Mater..

[4] Jiang K., Huang Y., Zeng G., Toma F. M., Goddard W. A., Bell A. T. (2020). Effects of Surface
Roughness on the Electrochemical
Reduction of CO over Cu. ACS Energy Letters.

[5] Arminio-Ravelo J. A., Favero S., Escudero-Escribano M. (2025). Why Testing
Protocols Matter in Electrochemical
Methane Oxidation: Insights from IrO_
*x*
_ in
Acid. ACS Energy Letters.

[6] Johnson A. (2025). Electrochemical
Surface Area (ECSA) Evaluation in Electrocatalysis: Principles, Measurement
Techniques, and Future Perspectives. Journal
of Engineering in Industrial Research.

[7] Hong M., Jo A. (2025). Improved electrochemical
surface area measurement of platinum using
a potential holding strategy. Electrochim. Acta.

[8] Anantharaj S., Karthik P. E., Noda S. (2023). Ambiguities
and best practices in
the determination of active sites and real surface area of monometallic
electrocatalytic interfaces. J. Colloid Interface
Sci..

[9] Xie X., Holze R. (2022). Electrode
Kinetic Data: Geometric vs. Real Surface Area. Batteries.

[10] Wei C., Sun S., Mandler D., Wang X., Qiao S. Z., Xu Z. J. (2019). Approaches
for measuring the surface areas of metal oxide electrocatalysts for
determining their intrinsic electrocatalytic activity. Chem. Soc. Rev..

[11] Łukaszewski M., Soszko M., Czerwiński A. (2016). Electrochemical Methods of Real Surface
Area Determination of Noble Metal Electrodes – an Overview. Int. J. Electrochem. Sci..

[12] Trasatti S., Petrii O. A. (1991). Real Surface Area
Measurements in Electrochemistry. Pure Appl.
Chem..

[13] Rudi S., Cui C., Gan L., Strasser P. (2014). Comparative Study of the Electrocatalytically
Active Surface Areas (ECSAs) of Pt Alloy Nanoparticles Evaluated by
Hupd and CO-stripping voltammetry. Electrocatalysis.

[14] Zhang B., Wang W., Liu C., Han L., Peng J., Oleinick A., Svir I., Amatore C., Tian Z., Zhan D. (2021). Surface Diffusion of Underpotential-Deposited
Lead Adatoms on Gold
Nanoelectrodes. ChemElectroChem..

[15] van
der Vliet D. F., Wang C., Li D., Paulikas A. P., Greeley J., Rankin R. B., Strmcnik D., Tripkovic D., Markovic N. M., Stamenkovic V. R. (2012). Unique Electrochemical Adsorption
Properties of Pt-Skin Surfaces. Angew. Chem.,
Int. Ed..

[16] Martínez-Hincapié R., Wegner J., Anwar M. U., Raza-Khan A., Franzka S., Kleszczynski S., Čolić V. (2024). The determination
of the electrochemically active surface area and its effects on the
electrocatalytic properties of structured nickel electrodes produced
by additive manufacturing. Electrochim. Acta.

[17] Röttcher N. C., Ku Y.-P., Minichova M., Ehelebe K., Cherevko S. (2023). Comparison
of methods to determine electrocatalysts’ surface area in gas
diffusion electrode setups: a case study on Pt/C and PtRu/C. Journal of Physics: Energy.

[18] Cossar E., Houache M. S., Zhang Z., Baranova E. A. (2020). Comparison
of electrochemical
active surface area methods for various nickel nanostructures. J. Electroanal. Chem..

[19] Sebastián-Pascual P., Jordão Pereira I., Escudero-Escribano M. (2020). Tailored electrocatalysts
by controlled electrochemical deposition and surface nanostructuring. Chem. Commun..

[20] Krupski K., Moors M., Jóźwik P., Kobiela T., Krupski A. (2015). Structure Determination of Au on Pt(111) Surface: LEED,
STM and DFT Study. Materials.

[21] Monteiro M. C., Koper M. T. (2019). Alumina contamination
through polishing and its effect
on hydrogen evolution on gold electrodes. Electrochim.
Acta.

[22] Sebastián-Pascual P., Escudero-Escribano M. (2020). Addressing
the Interfacial Properties for CO Electroreduction
on Cu with Cyclic Voltammetry. ACS Energy Letters.

[23] Sebastián-Pascual P., Petersen A. S., Bagger A., Rossmeisl J., Escudero-Escribano M. (2021). pH and Anion
Effects on Cu–Phosphate Interfaces
for CO Electroreduction. ACS Catal..

[24] Couce P. M., Madsen T. K., Plaza-Mayoral E., Kristoffersen H. H., Chorkendorff I., Dalby K. N., Van Der
Stam W., Rossmeisl J., Escudero-Escribano M., Sebastián-Pascual P. (2024). Tailoring
the facet distribution on copper with chloride. Chemical Science.

[25] Topalov A. A., Cherevko S., Zeradjanin A. R., Meier J. C., Katsounaros I., Mayrhofer K. J. J. (2014). Towards a comprehensive understanding of platinum dissolution
in acidic media. Chem. Sci..

[26] Valls
Mascaró F., McCrum I. T., Koper M. T. M., Rost M. J. (2022). Nucleation
and Growth of Dendritic Islands during Platinum Oxidation-Reduction
Cycling. J. Electrochem. Soc..

[27] Moser T., Valls Mascaró F., Kunze-Liebhäuser J. (2025). Dynamics of Adatom
and Vacancy Islands on Au(111) in Alkaline and Acidic Media. J. Phys. Chem. C.

[28] Kibler L., Cuesta A., Kleinert M., Kolb D. (2000). In-situ STM
characterisation
of the surface morphology of platinum single crystal electrodes as
a function of their preparation. J. Electroanal.
Chem..

[29] Rost M. J., Van Gastel R., Frenken J. W. M. (2000). Anomalous Shape and Decay of Islands
on Au(110). Phys. Rev. Lett..

[30] Van
Albada S. B., Rost M. J., Frenken J. W. M. (2002). Asymmetric and
symmetric Wulff constructions of island shapes on a missing-row reconstructed
surface. Phys. Rev. B.

[31] Valls
Mascaró F., Koper M. T. M., Rost M. J. (2024). Step bunching instability
and its effects in electrocatalysis on platinum surfaces. Nature Catalysis.

[32] Rosenfeld G., Morgenstern K., Esser M., Comsa G. (1999). Dynamics and
stability
of nanostructures on metal surfaces. Applied
Physics A: Materials Science & Processing.

[33] Morgenstern K., Rosenfeld G., Comsa G. (1996). Decay of Two-Dimensional Ag Islands
on Ag(111). Phys. Rev. Lett..

[34] Wulff G. (1901). XXV. Zur Frage
der Geschwindigkeit des Wachsthums und der Auflösung der Krystallflächen. Zeitschrift für Kristallographie - Crystalline Materials.

[35] Cuenya B. R. (2010). Synthesis
and catalytic properties of metal nanoparticles: Size, shape, support,
composition, and oxidation state effects. Thin
Solid Films.

[36] Sedano
Varo E., Egeberg Tankard R., Kryger-Baggesen J., Jinschek J., Helveg S., Chorkendorff I., Damsgaard C. D., Kibsgaard J. (2024). Gold Nanoparticles for CO Electroreduction:
An Optimum Defined by Size and Shape. J. Am.
Chem. Soc..

[37] Buffat P., Borel J.-P. (1976). Size effect on the melting temperature of gold particles. Phys. Rev. A.

[38] Watzele S., Hauenstein P., Liang Y., Xue S., Fichtner J., Garlyyev B., Scieszka D., Claudel F., Maillard F., Bandarenka A. S. (2019). Determination of Electroactive Surface
Area of Ni-,
Co-, Fe-, and Ir-Based Oxide Electrocatalysts. ACS Catal..

[39] Do U. P., Seland F., Johannessen E. A. (2018). The Real Area of Nanoporous Catalytic
Surfaces of Gold and Palladium in Aqueous Solutions. J. Electrochem. Soc..

[40] Meyer, E. ; Bennewitz, R. ; Hug, H. J. Scanning Probe Microscopy: The Lab on a Tip; Graduate Texts in Physics; Springer International Publishing: Cham, 2021.

[41] Cignoni P., Hosseini P., Kaiser C., Trost O., Nettler D.-R., Trzebiatowski L., Tschulik K. (2023). Validating Electrochemical Active
Surface Area Determination of Nanostructured Electrodes: Surface Oxide
Reduction on AuPd Nanoparticles. J. Electrochem.
Soc..

[42] Oswald S., Pritzl D., Wetjen M., Gasteiger H. A. (2020). Novel Method
for Monitoring the Electrochemical Capacitance by In Situ Impedance
Spectroscopy as Indicator for Particle Cracking of Nickel-Rich NCMs:
Part I. Theory and Validation. J. Electrochem.
Soc..

[43] Rouquerol, J. ; Rouquerol, F. ; Llewellyn, P. ; Maurin, G. ; Sing, K. S. W. Adsorption by Powders and Porous Solids: Principles, Methodology and Applications, 2nd ed.; Academic Press: Oxford, 2014.

[44] Leach, R. Optical Measurement of Surface Topography; Springer: Berlin, Heidelberg, 2011.

[45] Fujiwara, H. Spectroscopic Ellipsometry Principles and Applications; John Wiley & Sons, 2007.

[46] Chorkendorff, I. ; Niemantsverdriet, J. W. Concepts of Modern Catalysis and Kinetics, 3rd ed.; Wiley, 2017.

[47] Roy C., Sebok B., Scott S. B., Fiordaliso E. M., Sørensen J. E., Bodin A., Trimarco D. B., Damsgaard C. D., Vesborg P. C. K., Hansen O., Stephens I. E. L., Kibsgaard J., Chorkendorff I. (2018). Impact of nanoparticle size and lattice oxygen on water
oxidation on NiFeOxHy. Nature Catalysis.

[48] Escudero-Escribano M., Malacrida P., Hansen M. H., Vej-Hansen U. G., Velázquez-Palenzuela A., Tripkovic V., Schiøtz J., Rossmeisl J., Stephens I. E. L., Chorkendorff I. (2016). Tuning the
activity of Pt alloy electrocatalysts by means of the lanthanide contraction. Science.

[49] Conway, B. E. Electrochemical Supercapacitors: Scientific Fundamentals and Technological Applications, 1st ed.; Springer: Boston, MA, 1999.

[50] Schmickler, W. ; Santos, E. Interfacial electrochemistry, 2nd ed.; Springer: Heidelberg; New York, 2010.

[51] Boo H., Park S., Ku B., Kim Y., Park J. H., Kim H. C., Chung T. D. (2004). Ionic Strength-Controlled
Virtual
Area of Mesoporous Platinum Electrode. J. Am.
Chem. Soc..

[52] Henrique F., Zuk P. J., Gupta A. (2021). Charging dynamics
of electrical double
layers inside a cylindrical pore: predicting the effects of arbitrary
pore size. Soft Matter.

[53] Morales D. M., Risch M. (2021). Seven steps to reliable
cyclic voltammetry measurements for the determination
of double layer capacitance. Journal of Physics:
Energy.

[54] Yoon Y., Yan B., Surendranath Y. (2018). Suppressing
Ion Transfer Enables Versatile Measurements
of Electrochemical Surface Area for Intrinsic Activity Comparisons. J. Am. Chem. Soc..

[55] Córdoba-Torres P., Mesquita T. J., Nogueira R. P. (2015). Relationship between the Origin of
Constant-Phase Element Behavior in Electrochemical Impedance Spectroscopy
and Electrode Surface Structure. J. Phys. Chem.
C.

[56] Lazanas A.
C., Prodromidis M. I. (2023). Electrochemical
Impedance Spectroscopy—A Tutorial. ACS
Measurement Science Au.

[57] Schalenbach M., Selmert V., Kretzschmar A., Raijmakers L., Durmus Y. E., Tempel H., Eichel R.-A. (2024). How microstructures,
oxide layers, and charge transfer reactions influence double layer
capacitances. Part 1: impedance spectroscopy and cyclic voltammetry
to estimate electrochemically active surface areas (ECSAs). Phys. Chem. Chem. Phys..

[58] Schalenbach M., Raijmakers L., Tempel H., Eichel R. (2025). How Microstructures,
Oxide Layers, and Charge Transfer Reactions Influence Double Layer
Capacitances. Part 2: Equivalent Circuit Models. Electrochemical Science Advances.

[59] Escudero-Escribano M., Verdaguer-Casadevall A., Malacrida P., Grønbjerg U., Knudsen B. P., Jepsen A. K., Rossmeisl J., Stephens I. E. L., Chorkendorff I. (2012). Pt_5_ Gd as a Highly Active
and Stable Catalyst for Oxygen Electroreduction. J. Am. Chem. Soc..

[60] Samjeské G., Komatsu K.-i., Osawa M. (2009). Dynamics of CO Oxidation on a Polycrystalline
Platinum Electrode: A Time-Resolved Infrared Study. J. Phys. Chem. C.

[61] Song K., Schneider P. M., Grabovac I., Garlyyev B., Watzele S. A., Bandarenka A. S. (2025). Influence of the Electrolyte pH on the Double Layer
Capacitance of Polycrystalline Pt and Au Electrodes in Acidic Solutions. ChemElectroChem..

[62] Mascaró F. V., Koper M. T., Rost M. J. (2024). Quantitative study of electrochemical
adsorption and oxidation on Pt(111) and its vicinal surfaces. Electrochim. Acta.

[63] Rizo R., Fernández-Vidal J., Hardwick L. J., Attard G. A., Vidal-Iglesias F. J., Climent V., Herrero E., Feliu J. M. (2022). Investigating
the presence of adsorbed species on Pt steps at low potentials. Nat. Commun..

[64] Chen X., Ojha K., Koper M. T. M. (2024). Deconvolution
of the Voltammetric
Features of a Pt(100) Single-Crystal Electrode. J. Phys. Chem. Lett..

[65] Strmcnik D., Escudero-Escribano M., Kodama K., Stamenkovic V. R., Cuesta A., Marković N. M. (2010). Enhanced electrocatalysis of the
oxygen reduction reaction based on patterning of platinum surfaces
with cyanide. Nat. Chem..

[66] Chen X., McCrum I. T., Schwarz K. A., Janik M. J., Koper M. T. M. (2017). Co-adsorption
of Cations as the Cause of the Apparent pH Dependence of Hydrogen
Adsorption on a Stepped Platinum Single-Crystal Electrode. Angew. Chem., Int. Ed..

[67] Madry B., Wandelt K., Nowicki M. (2016). Deposition of copper and sulfate
on Au(111): New insights. Appl. Surf. Sci..

[68] Nettler D.-R., Clausmeyer J., Savan A., Cignoni P., Rurainsky C., Drautz R., Ludwig A., Tschulik K. (2025). Does Pb underpotential
deposition rearrange surface-near atoms in AgAu films and nanoparticles?. Electrochim. Acta.

[69] Gómez R., Feliu J., Aldaz A., Weaver M. (1998). Validity of double-layer
charge-corrected voltammetry for assaying carbon monoxide coverages
on ordered transition metals: comparisons with adlayer structures
in electrochemical and ultrahigh vacuum environments. Surf. Sci..

[70] Ochal P., Gomez De La Fuente J.
L., Tsypkin M., Seland F., Sunde S., Muthuswamy N., Rønning M., Chen D., Garcia S., Alayoglu S., Eichhorn B. (2011). CO stripping
as an electrochemical tool for characterization of Ru@Pt core-shell
catalysts. J. Electroanal. Chem..

[71] Jacobse L., Huang Y.-F., Koper M. T. M., Rost M. J. (2018). Correlation of surface
site formation to nanoisland growth in the electrochemical roughening
of Pt(111). Nat. Mater..

[72] Deng X., Galli F., Koper M. T. (2020). In Situ
AFM Imaging of Platinum Electrode
Surface during Oxidation–Reduction Cycles in Alkaline Electrolyte. ACS Applied Energy Materials.

[73] Behjati S., Koper M. T. M. (2024). In Situ STM Study
of Roughening of Au(111) Single-Crystal
Electrode in Sulfuric Acid Solution during Oxidation–Reduction
Cycles. J. Phys. Chem. C.

[74] Zhou Y., Bowers B., Bagger A., Yang G., Steier L., Ryan M. P., Stephens I. E. L. (2025). Revisiting
Active Site Quantification
in CO Electroreduction: The Case for CO Displacement. ACS Energy Letters.

[75] Malko D., Kucernak A., Lopes T. (2016). In situ electrochemical
quantification
of active sites in Fe–N/C non-precious metal catalysts. Nat. Commun..

[76] Frumkin, A. N. Advances in Electrochemistry and Electrochemical Engineering; Interscience Publishers: p315, 1963; Vol. 3; pp 287–391.

[77] Brummer S. B. (1965). The Use
of Large Anodic Galvanostatic Transients to Evaluate the Maximum Adsorption
on Platinum from Formic Acid Solutions. J. Phys.
Chem..

[78] Beden B., Floner D., Léger J., Lamy C. (1985). A voltammetric study
of the formation of hydroxides and oxyhydroxides on nickel single
crystal electrodes in contact with an alkaline solution. Surface Science Letters.

[79] Ganassin A., Sebastián P., Climent V., Schuhmann W., Bandarenka A. S., Feliu J. (2017). On the pH Dependence of the Potential
of Maximum Entropy of Ir(111) Electrodes. Sci.
Rep..

[80] Sebastián-Pascual P., Escudero-Escribano M. (2021). Surface characterization
of copper electrocatalysts
by lead underpotential deposition. J. Electroanal.
Chem..

[81] Hochfilzer D., Tiwari A., Clark E. L., Bjørnlund A. S., Maagaard T., Horch S., Seger B., Chorkendorff I., Kibsgaard J. (2022). *In* Situ Analysis
of the Facets of
Cu-Based Electrocatalysts in Alkaline Media Using Pb Underpotential
Deposition. Langmuir.

[82] Schmidt T. O., Ngoipala A., Arevalo R. L., Watzele S. A., Lipin R., Kluge R. M., Hou S., Haid R. W., Senyshyn A., Gubanova E. L., Bandarenka A. S., Vandichel M. (2022). Elucidation
of Structure–Activity Relations in Proton Electroreduction
at Pd Surfaces: Theoretical and Experimental Study. Small.

[83] Tan Z., Li K., Gu Y., Nan Z., Wang W., Sun L., Mao B., Yan J. (2023). Unconventional
Electrochemical Behaviors of Cu Underpotential
Deposition in a Chloride-Based Deep Eutectic Solvent: High Underpotential
Shift and Low Coverage. Anal. Chem..

[84] Green C. L., Kucernak A. (2002). Determination of the
Platinum and Ruthenium Surface
Areas in Platinum–Ruthenium Alloy Electrocatalysts by Underpotential
Deposition of Copper. I. Unsupported Catalysts. J. Phys. Chem. B.

[85] Stephens I. E. L., Bondarenko A. S., Perez-Alonso F. J., Calle-Vallejo F., Bech L., Johansson T. P., Jepsen A. K., Frydendal R., Knudsen B. P., Rossmeisl J., Chorkendorff I. (2011). Tuning the Activity of Pt(111) for Oxygen Electroreduction
by Subsurface Alloying. J. Am. Chem. Soc..

[86] Jacobse L., Vonk V., McCrum I. T., Seitz C., Koper M. T., Rost M. J., Stierle A. (2022). Electrochemical
oxidation of Pt(111)
beyond the place-exchange model. Electrochim.
Acta.

[87] Oswald S., Pritzl D., Wetjen M., Gasteiger H. A. (2021). Novel Method
for Monitoring the Electrochemical Capacitance by In Situ Impedance
Spectroscopy as Indicator for Particle Cracking of Nickel-Rich NCMs:
Part II. Effect of Oxygen Release Dependent on Particle Morphology. J. Electrochem. Soc..

[88] Reiser C., Keßler P., Kamp M., Jovic V., Moser S. (2023). Specific Capacitance
of RuO_2_ (110) Depends Sensitively on Surface Order. J. Phys. Chem. C.

[89] Connor P., Schuch J., Kaiser B., Jaegermann W. (2020). The Determination
of Electrochemical Active Surface Area and Specific Capacity Revisited
for the System MnO_x_ as an Oxygen Evolution Catalyst. Zeitschrift für Physikalische Chemie.

[90] Sjö H., Shabalin A., Lienert U., Hektor J., Schaefer A., Carlsson P.-A., Alwmark C., Gustafson J. (2025). Surface grain
orientation mapping using grazing incidence X-ray diffraction. Surf. Sci..

[91] Jacobse L., Rost M. J., Koper M. T. M. (2019). Atomic-Scale Identification of the
Electrochemical Roughening of Platinum. ACS
Central Science.

[92] Clavilier J., El Achi K., Rodes A. (1989). In situ characterization
of the Pt­(S)-[n(111)
/sx (111)] electrode surfaces using electrosorbed hydrogen for probing
terrace an step sites. Journal of Electroanalytical
Chemistry and Interfacial Electrochemistry.

[93] Clavilier J., El Achi K., Rodes A. (1990). In situ probing
of step and terrace
sites on Pt­(S)-[n(111) × (111)] electrodes. Chem. Phys..

[94] Jiang H., He Q., Zhang Y., Song L. (2018). Structural Self-Reconstruction of
Catalysts in Electrocatalysis. Acc. Chem. Res..

[95] Hermann J. M., Abdelrahman A., Jacob T., Kibler L. A. (2020). Potential-dependent
reconstruction kinetics probed by HER on Au(111) electrodes. Electrochim. Acta.

[96] Iizuka K., Kumeda T., Suzuki K., Tajiri H., Sakata O., Hoshi N., Nakamura M. (2022). Tailoring the active site for the
oxygen evolution reaction on a Pt electrode. Communications Chemistry.

