# Supplementary material for: The Hidden Complexities of Electrochemically Active Surface Area Measurements
Source: ACS Energy Lett. 2026 Feb 11;11(3):2508–20. doi: 10.1021/acsenergylett.5c04204 (PMC13007280; doi:10.1021/acsenergylett.5c04204)
Supplement: Supplementary file 1 [file nz5c04204_si_001.pdf]

Supporting Information: The Hidden Complexities of Electrochemically Active Surface Area Measurements

Jon Bjarke Valbæk Mygind,*,† Marcel J. Rost,‡ and María Escudero-Escribano*,†,¶

†*Catalan Institute of Nanoscience and Nanotechnology, Campus UAB, Bellaterra, 08193 Barcelona, Spain*

‡*Huygens-Kamerlingh Onnes Laboratory, Leiden University, Niels Bohrweg 2, 2333 CA Leiden, The Netherlands*

¶*Catalan Institution for Research and Advanced Studies (ICREA), Passeig de Lluís Companys, 23, 08010 Barcelona, Spain*

E-mail: jon.mygind@icn2.cat; maria.escudero@icn2.cat

Contents

1 Notation **S-4**

2 Real Surface Area **S-4**

2.1 Domain Distribution of the Real Surface Area S-4

2.2 Surface-specific capacitance from domain contributions S-5

2.3 Real surface area from capacitance S-5

2.4 Surface-specific charge from domain contributions S-6

2.5 Real surface area from charge S-6

2.6 Real surface area from domain-resolved charge fractions S-6

2.7 Real surface area from domain charge fractions S-7

3 Electrochemically Active Surface Area **S-8**

3.1 ECSA from coverage S-8

3.2 Surface units for unit-charge construction S-9

3.3 Coverage from unit charge S-10

3.4 ECSA using domain-specific RSA S-10

3.5 ECSA via domain specific charge fractions S-10

4 Active Site Count **S-11**

5 Case Study on Worked Example **S-12**

5.1 RSA Determination S-13

5.2 ECSA Determination S-15

5.2.1 Cu(111) Unit Charge S-18

5.2.2 Cu(110) Unit Charge S-18

5.2.3 Cu(100) Unit Charge S-19

5.2.4 Cu(310) Unit Charge S-19

5.2.5 Computing the ECSA S-20
5.3 Active Site Count Determination S-22
5.4 Normalization to Electrocatalytic Current S-23

References **S-24**

1 Notation

Throughout this Supporting Information, quantities measured on the sample electrode carry the superscript (sample), while quantities measured on domain-resolved reference electrodes carry the superscript (ref). Superscripts encode only the identity of the electrode from which a quantity is obtained.

Subscripts indicate the physical meaning of a quantity and, when applicable, the domain index i.

2 Real Surface Area

The real surface area (RSA) denotes the total geometric area of the electrode, including all terraces, steps, kinks, and other microscopic features. It is the three-dimensional interfacial area that would be measured if the surface topography were fully resolved, and it generally differs from the projected two-dimensional geometric area.

2.1 Domain Distribution of the Real Surface Area

A real electrode consists of multiple surface domains, each characterized by a distinct atomic arrangement. RSA of the sample electrode is therefore the sum of the real surface areas associated with each domain. For a domain of type i, the RSA contributed by that domain is denoted $A_{\mathrm{RSA},i}^{(\mathrm{sample})}$. The total RSA of the sample is given by Equation 1.

$$A_{\mathrm{RSA}}^{(\mathrm{sample})} = \sum_i A_{\mathrm{RSA},i}^{(\mathrm{sample})} \tag{1}$$

The fractional RSA associated with each domain is defined in Equation 2.

$$x_{\mathrm{RSA},i} = \frac{A_{\mathrm{RSA},i}^{(\mathrm{sample})}}{A_{\mathrm{RSA}}^{(\mathrm{sample})}}, \qquad \sum_i x_{\mathrm{RSA},i} = 1 \tag{2}$$

For each domain i, the corresponding RSA measured on a single-crystal reference electrode exposing only that domain is denoted $A^{(\mathrm{ref})}_{\mathrm{RSA},i}$.

2.2 Surface-specific capacitance from domain contributions

We assume that domain i exhibits the same surface-specific double-layer capacitance in the sample and in the corresponding reference measurement. This relation is given in Equation 3.

$$\frac{C_i^{(\mathrm{sample})}}{A^{(\mathrm{sample})}_{\mathrm{RSA},i}} = \frac{C_i^{(\mathrm{ref})}}{A^{(\mathrm{ref})}_{\mathrm{RSA},i}} \tag{3}$$

Multiplying both sides by the area fraction $x_{\mathrm{RSA},i}$ gives the expression in Equation 4.

$$\frac{C_i^{(\mathrm{sample})}}{A^{(\mathrm{sample})}_{\mathrm{RSA}}} = x_{\mathrm{RSA},i}\,\frac{C_i^{(\mathrm{ref})}}{A^{(\mathrm{ref})}_{\mathrm{RSA},i}} \tag{4}$$

Summing over all domains yields the sample's surface-specific capacitance, as shown in Equation 5.

$$C_{\mathrm{s}}^{(\mathrm{sample})} = \sum_i \frac{C_i^{(\mathrm{sample})}}{A^{(\mathrm{sample})}_{\mathrm{RSA}}} = \sum_i x_{\mathrm{RSA},i}\,\frac{C_i^{(\mathrm{ref})}}{A^{(\mathrm{ref})}_{\mathrm{RSA},i}} \tag{5}$$

2.3 Real surface area from capacitance

The total double-layer capacitance measured for the sample, $C^{(\mathrm{sample})}$, must equal the product of its real surface area and its surface-specific capacitance. Thus, the surface-specific capacitance can be described, as shown in Equation 6.

$$A^{(\mathrm{sample})}_{\mathrm{RSA}} = \frac{C^{(\mathrm{sample})}}{C_{\mathrm{s}}^{(\mathrm{sample})}} \tag{6}$$

Inserting Equation 5 into Equation 6 gives the expression shown in Equation 7.

$$A_{\mathrm{RSA}}^{(\mathrm{sample})} = \frac{C^{(\mathrm{sample})}}{\sum_i x_{\mathrm{RSA},i} \frac{C_i^{(\mathrm{ref})}}{A_{\mathrm{RSA},i}^{(\mathrm{ref})}}}. \tag{7}$$

2.4 Surface-specific charge from domain contributions

Because the sample electrode contains a distribution of domains, the surface-specific charge associated with an adsorption-limited faradaic probe reaction is obtained as a domain-weighted combination of reference surface-specific charges, analogous to Equation 5.

$$Q_{\mathrm{s}}^{(\mathrm{sample})} = \sum_i x_{\mathrm{RSA},i} \frac{Q_i^{(\mathrm{ref})}}{A_{\mathrm{RSA},i}^{(\mathrm{ref})}} \tag{8}$$

2.5 Real surface area from charge

For an adsorption-limited faradaic probe reaction, the corrected faradaic charge measured for the sample, $Q^{(\mathrm{sample})}$, must equal the product of its real surface area and its surface-specific faradaic charge. Thus, the surface-specific charge can be described, as shown in Equation 9.

$$A_{\mathrm{RSA}}^{(\mathrm{sample})} = \frac{Q^{(\mathrm{sample})}}{Q_{\mathrm{s}}^{(\mathrm{sample})}} \tag{9}$$

Inserting Equation 8 into Equation 9 yields the expression given in Equation 10.

$$A_{\mathrm{RSA}}^{(\mathrm{sample})} = \frac{Q^{(\mathrm{sample})}}{\sum_i x_{\mathrm{RSA},i} \frac{Q_i^{(\mathrm{ref})}}{A_{\mathrm{RSA},i}^{(\mathrm{ref})}}} \tag{10}$$

2.6 Real surface area from domain-resolved charge fractions

Adsorption-limited faradaic reactions can exhibit distinct, domain-specific features in the voltammetric response. When these features are well separated in potential, the total

faradaic charge of the sample electrode can be decomposed into contributions associated with individual domains. For a domain of type i, the contribution to the total charge of the sample is denoted $Q_i^{(\text{sample})}$. The total charge of the sample is given in the expression below.

$$Q^{(\text{sample})} = \sum_i Q_i^{(\text{sample})} \tag{11}$$

The fractional charge associated with each domain is defined in Equation 12.

$$x_{\text{Charge},i} = \frac{Q_i^{(\text{sample})}}{Q^{(\text{sample})}}, \qquad \sum_i x_{\text{Charge},i} = 1 \tag{12}$$

For each domain i, the corresponding charge measured on a single-crystal reference electrode exposing only that domain is denoted $Q_i^{(\text{ref})}$.

2.7 Real surface area from domain charge fractions

We now relate the domain-resolved charge contributions of the sample to its domain-resolved real surface areas. We assume that domain i exhibits the same surface-specific adsorption-limited faradaic charge in the sample as in the corresponding reference measurement. This relation is given in Equation 13.

$$\frac{Q_i^{(\text{sample})}}{A_{\text{RSA},i}^{(\text{sample})}} = \frac{Q_i^{(\text{ref})}}{A_{\text{RSA},i}^{(\text{ref})}} \tag{13}$$

Rearranging this relation gives the real surface area of domain i in the sample.

$$A_{\text{RSA},i}^{(\text{sample})} = Q_i^{(\text{sample})} \frac{A_{\text{RSA},i}^{(\text{ref})}}{Q_i^{(\text{ref})}} \tag{14}$$

The total RSA of the sample is obtained by summing the contributions from all domains.

$$A_{\text{RSA}}^{(\text{sample})} = \sum_i A_{\text{RSA},i}^{(\text{sample})} = \sum_i Q_i^{(\text{sample})} \frac{A_{\text{RSA},i}^{(\text{ref})}}{Q_i^{(\text{ref})}}. \tag{15}$$

The domain-resolved charge contribution of domain i may be written in terms of the domain-specific charge fraction of domain i combined with the total charge of the sample.

$$Q_i^{(\mathrm{sample})} = x_{\mathrm{Charge},i}\, Q^{(\mathrm{sample})} \tag{16}$$

Substituting Equation. 16 into Equation. 15 gives the following expression for the RSA of the sample.

$$A_{\mathrm{RSA}}^{(\mathrm{sample})} = \sum_i x_{\mathrm{Charge},i}\, Q^{(\mathrm{sample})} \left(\frac{A_{\mathrm{RSA},i}^{(\mathrm{ref})}}{Q_i^{(\mathrm{ref})}} \right) \tag{17}$$

3 Electrochemically Active Surface Area

The electrochemically active surface area (ECSA) is the subset of the real surface area that participates in a specific faradaic reaction under defined operational conditions.

3.1 ECSA from coverage

For an adsorption-limited faradaic probe reaction, the ECSA is obtained from RSA through the coverage of the probe species, θ_{probe}.

$$A_{\mathrm{ECSA}} = \theta_{\mathrm{probe}}\, A_{\mathrm{RSA}} \tag{18}$$

The probe coverage represents the fraction of surface sites that participate in the probe reaction under the measurement conditions, and is defined as:

$$\theta_{\mathrm{probe}} = \frac{N_{\mathrm{active}}}{N_{\mathrm{tot}}} \tag{19}$$

where N_{active} is the number of probe-occupied sites and N_{tot} is the total number of probe-accessible sites on the corresponding surface. For a sample electrode containing multiple domains, the probe coverage may differ across domains. We therefore define a domain-

specific coverage θ_i for each domain type i. The ECSA associated with that domain is given by Equation 20.

$$A_{\mathrm{ECSA},i} = \theta_{\mathrm{probe},i}\, A_{\mathrm{RSA},i} \tag{20}$$

Summing over all domains yields the total ECSA as described in Equation 21.

$$A_{\mathrm{ECSA}}^{(\mathrm{sample})} = \sum_i \theta_{\mathrm{probe},i}\, A_{\mathrm{RSA},i}^{(\mathrm{sample})} \tag{21}$$

Although a domain may contain multiple distinct adsorption sites, these sites are not necessarily independent; occupation of one site type can block or modify access to neighbouring sites. Because the probe-relevant adsorption configuration is usually unknown, and because individual site types may overlap spatially, we therefore use a domain-averaged coverage θ_i rather than a site-resolved decomposition. Importantly, θ_i is an effective quantity that captures the average probe response of a given domain under the measurement conditions. It does not imply that all nominally equivalent sites within a domain are equally active or equally accessible.

3.2 Surface units for unit-charge construction

To evaluate the coverage on each domain of the sample, we must determine the maximum site density accessible to the probe reaction. This is obtained by constructing a surface unit: a periodic or otherwise representative surface cell containing the probe-relevant adsorption sites. The unit area $A_{\mathrm{unit},i}$ is simply the RSA of the defined periodic surface unit in domain i. The unit charge $Q_{\mathrm{unit},i}$ of domain i per unit surface area is then:

$$\frac{Q_{\mathrm{unit},i}}{A_{\mathrm{unit},i}} = \frac{N_{\mathrm{sites},i}\, n_{\mathrm{probe}}\, e}{A_{\mathrm{unit},i}} \tag{22}$$

where $N_{\mathrm{sites},i}$ is the number of probe relevant adsorption sites, n_{probe} is the number of electrons transferred per adsorbed species, and e is the elementary charge.

3.3 Coverage from unit charge

The coverage of the probe species on domain i is obtained by comparing the measured charge per RSA with the corresponding unit charge per unit area. Because the charge per surface area of domain i of the sample equals that of the reference, the coverage can be expressed as shown in Equation 23.

$$\theta_{\text{probe},i} = \frac{\dfrac{Q_i^{(\text{sample})}}{A_{\text{RSA},i}^{(\text{sample})}}}{\dfrac{Q_{\text{unit},i}}{A_{\text{unit},i}}} = \frac{\dfrac{Q_i^{(\text{ref})}}{A_{\text{RSA},i}^{(\text{ref})}}}{\dfrac{Q_{\text{unit},i}}{A_{\text{unit},i}}} \tag{23}$$

An equivalent expression for the coverage is given in Equation 24.

$$\theta_{\text{probe},i} = \frac{Q_i^{(\text{sample})}}{A_{\text{RSA},i}^{(\text{sample})}} \left(\frac{A_{\text{unit},i}}{Q_{\text{unit},i}} \right) = \frac{Q_i^{(\text{ref})}}{A_{\text{RSA},i}^{(\text{ref})}} \left(\frac{A_{\text{unit},i}}{Q_{\text{unit},i}} \right) \tag{24}$$

3.4 ECSA using domain-specific RSA

To calculate the ECSA of the sample we can insert the expression from Equation 24 into Equation 21

$$A_{\text{ECSA}}^{(\text{sample})} = \sum_i Q_i^{(\text{ref})} \frac{A_{\text{RSA},i}^{(\text{sample})}}{A_{\text{RSA},i}^{(\text{ref})}} \left(\frac{A_{\text{unit},i}}{Q_{\text{unit},i}} \right) \tag{25}$$

provided that we know the RSA domain distribution of the sample.

3.5 ECSA via domain specific charge fractions

If the domain-specific charge fractions are known, the ECSA can be obtained by inserting the coverage expression from Equation 24 into Equation 21 and using the charge decomposition given in Equation 16. The resulting expression is shown in Equation 49.

$$A_{\mathrm{ECSA}}^{(\mathrm{sample})} = \sum_{i} x_{\mathrm{Charge},i}\, Q^{(\mathrm{sample})} \left(\frac{A_{\mathrm{unit},i}}{Q_{\mathrm{unit},i}} \right) \tag{26}$$

4 Active Site Count

For an adsorption-limited faradaic probe reaction, the number of participating surface sites can be inferred directly from the measured charge, provided that the electron stoichiometry of the probe reaction is known and that non-probe-related charge contributions have been removed. This approach deliberately abandons any explicit description of surface geometry, periodicity, or site identity. Instead, the electrode surface is treated as a black box, and only the total number of electrons transferred in the probe reaction is considered.

Within this operational framework, the transfer of n_{probe} electrons corresponds to a single reaction event of the probe species. If the probe reaction is strictly surface-confined and self-limiting, the total number of reaction events can be related to an effective number of surface sites that participate under the probe conditions. Importantly, this is the same underlying assumption employed in unit-charge constructions, where discrete electron-transfer events are equated with adsorption events on a surface.

Sites whose activity depends strongly on local environment, coordination, or strain simply contribute proportionally less (or not at all) to the measured charge. The active-site-count formalism does not aim to distinguish between different site types or to assess their intrinsic activity; it provides an operational measure of how many sites participate in the probe reaction under the given conditions.

$$N_{\mathrm{active}}^{(\mathrm{sample})} = \frac{Q^{(\mathrm{sample})}}{e \cdot n_{\mathrm{probe}}} \tag{27}$$

5 Case Study on Worked Example

This section provides a worked example illustrating how the RSA, ECSA, and active-site count can be determined using experimentally accessible electrochemical probes. The workflow follows an example inspired by the study of Couce et al.,[S1] who employed Pb underpotential deposition (Pb-UPD) to resolve domain distribution of crystallographic orientations on a polycrystalline copper electrode. Their study serves here as a concrete reference case because Pb-UPD, under these conditions, generates distinct features that allow direct extraction of crystallographic contributions.. Figure S1 shows both the experimentally measured Pb-UPD response and the corresponding simulated voltammogram with its orientation-assigned peak components, which together illustrate how the deconvolution used in this example is constructed.

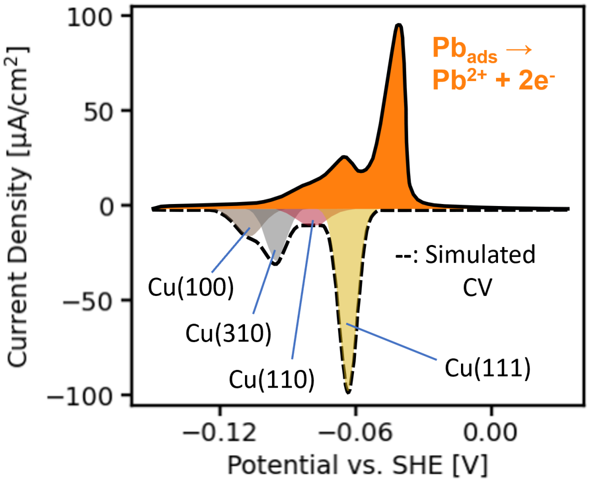

Figure S1: Illustration of the Pb-UPD stripping response on polycrystalline Cu, highlighting the characteristic peak shapes associated with the Cu(100), Cu(310), Cu(110), and Cu(111) crystallographic orientations. Cyclic voltammograms were recorded at 5 $mV\,s^{-1}$ in an electrolyte containing 0.1 M $KClO_4$ + 2 mM NaCl + 2 mM $Pb(ClO_4)_2$ + 1 mM $HClO_4$, using a three-electrode configuration with an SCE reference. The Cu(poly) electrode was electropolished prior to measurement. Data re-drawn from Couce et al.[S1]

Table S1 summarizes the measured Pb-UPD charges for representative Cu single-crystal electrodes and for the polycrystalline Cu electrode used in this example. For the poly-

crystalline sample, the charge associated with each domain-assigned potential region is also integrated. These charges are then normalized by the total Pb-UPD charge of the same electrode to obtain the domain-specific fractions.

Table S1: Geometric areas, Pb-UPD charges for Cu single crystals and polycrystalline Cu, and charge based domain fractions $x_{\mathrm{Charge},i}$ obtained from the deconvolution shown in Figure S1.

	Cu(poly)	Cu(111)	Cu(110)	Cu(100)	Cu(310)
A_{geo} (cm^2)	0.20 ± 0.01	0.20 ± 0.01	0.20 ± 0.01	0.20 ± 0.01	0.20 ± 0.01
Q_{corr} (μC)	73 ± 5	70 ± 2	68 ± 2	75 ± 4	64 ± 1
$x_{\mathrm{Charge,Cu(111)}}$	0.59 ± 0.03	—	—	—	—
$x_{\mathrm{Charge,Cu(110)}}$	0.09 ± 0.01	—	—	—	—
$x_{\mathrm{Charge,Cu(100)}}$	0.13 ± 0.02	—	—	—	—
$x_{\mathrm{Charge,Cu(310)}}$	0.19 ± 0.02	—	—	—	—

5.1 RSA Determination

To determine the RSA of the single-crystal electrodes, we first consider the low-index Cu surfaces. For Cu(111), Cu(110), and Cu(100), the macroscopic surface is cut parallel to the corresponding crystallographic plane. Under the assumption of an ideal, defect-free single crystal, neglecting thermally activated roughness and steps, the RSA equals the macroscopic geometric area.

$$A^{(\mathrm{ref})}_{\mathrm{RSA,Cu(111)}} = A^{(\mathrm{ref})}_{\mathrm{geo,Cu(111)}} = 0.20 \pm 0.01\,\mathrm{cm}^2 \tag{28}$$

$$A^{(\mathrm{ref})}_{\mathrm{RSA,Cu(110)}} = A^{(\mathrm{ref})}_{\mathrm{geo,Cu(110)}} = 0.20 \pm 0.01\,\mathrm{cm}^2 \tag{29}$$

$$A^{(\mathrm{ref})}_{\mathrm{RSA,Cu(100)}} = A^{(\mathrm{ref})}_{\mathrm{geo,Cu(100)}} = 0.20 \pm 0.01\,\mathrm{cm}^2 \tag{30}$$

In contrast, the Cu(310) surface is a vicinal plane composed of (100) terraces and (110)-type steps. Its macroscopic normal is tilted relative to that of the (100) plane by an angle

β. This angle follows from the scalar product of the corresponding plane normals, as shown in Equation 31.

$$\cos(\beta) = \frac{|(h_1, k_1, l_1) \cdot (h_2, k_2, l_2)|}{\sqrt{h_1^2 + k_1^2 + l_1^2}\,\sqrt{h_2^2 + k_2^2 + l_2^2}} = \cos(18.43°) \tag{31}$$

The geometric area $A^{(\mathrm{ref})}_{\mathrm{geo,Cu(310)}}$ corresponds to the projected area of the (310) plane and therefore does not represent the RSA. Using the tilt angle and basic trigonometry, the RSA of Cu(310) is given by Equation 32.

$$A^{(\mathrm{ref})}_{\mathrm{RSA,Cu(310)}} = A^{(\mathrm{ref})}_{\mathrm{geo,Cu(310)}}(\cos\beta + \sin\beta) = 0.25 \pm 0.01\,\mathrm{cm}^2 \tag{32}$$

This construction is illustrated in Figure S2, which shows a cross-sectional sketch of the Cu(310) surface. The RSA contribution of this vicinal surface can be decomposed into a terrace-aligned component ($\cos\beta$ term) and a step-wall component ($\sin\beta$ term), as shown by the red triangle. Together, these two components give the RSA.

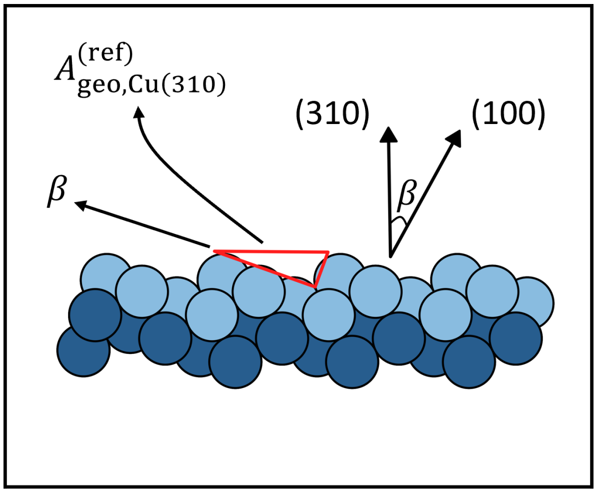

Figure S2: Cross-sectional schematic of the Cu(310) surface illustrating how the RSA exceeds the geometric area. The vicinal plane consists of (100) terraces and (110)-type steps, producing a tilted surface whose true area is obtained from the terrace and step-wall contributions.

The sample RSA follows directly from the domain-resolved charge fractions, as given in

Equation 33.

$$A_{\mathrm{RSA}}^{(\mathrm{sample})} = \sum_i x_{\mathrm{Charge},i}\, Q^{(\mathrm{sample})} \left(\frac{A_{\mathrm{RSA},i}^{(\mathrm{ref})}}{Q_i^{(\mathrm{ref})}} \right) \tag{33}$$

Inserting the domain-specific charge fractions and reference conversion factors into Equation 33 yields the expression shown in Equation 34.

$$\begin{aligned} A_{\mathrm{RSA,Cu(poly)}}^{(\mathrm{sample})} = \quad & x_{\mathrm{Charge,Cu(111)}}\, Q_{\mathrm{Cu(poly)}}^{(\mathrm{sample})} \left(\frac{A_{\mathrm{RSA,Cu(111)}}^{(\mathrm{ref})}}{Q_{\mathrm{Cu(111)}}^{(\mathrm{ref})}} \right) \\ & + x_{\mathrm{Charge,Cu(110)}}\, Q_{\mathrm{Cu(poly)}}^{(\mathrm{sample})} \left(\frac{A_{\mathrm{RSA,Cu(110)}}^{(\mathrm{ref})}}{Q_{\mathrm{Cu(110)}}^{(\mathrm{ref})}} \right) \\ & + x_{\mathrm{Charge,Cu(100)}}\, Q_{\mathrm{Cu(poly)}}^{(\mathrm{sample})} \left(\frac{A_{\mathrm{RSA,Cu(100)}}^{(\mathrm{ref})}}{Q_{\mathrm{Cu(100)}}^{(\mathrm{ref})}} \right) \\ & + x_{\mathrm{Charge,Cu(310)}}\, Q_{\mathrm{Cu(poly)}}^{(\mathrm{sample})} \left(\frac{A_{\mathrm{RSA,Cu(310)}}^{(\mathrm{ref})}}{Q_{\mathrm{Cu(310)}}^{(\mathrm{ref})}} \right) \end{aligned} \tag{34}$$

Using the numerical values in Table S1 and propagating the associated uncertainties following the first-order method of Ref.,[S2] we obtain the result shown in Equation 35.

$$A_{\mathrm{RSA,Cu(poly)}}^{(\mathrm{sample})} = 0.22 \pm 0.02\ \mathrm{cm}^2 \tag{35}$$

5.2 ECSA Determination

To determine the ECSA, we must construct unit charges per unit surface area for the probe reaction (i.e., Pb-UPD at full coverage). Pb-UPD involves a two-electron transfer reaction ($n_{\mathrm{probe}} = 2$). To decide whether the unit area should be based on the Cu lattice constant ($a_{\mathrm{Cu}} = 361.49, \mathrm{pm}$) or the Pb lattice constant ($a_{\mathrm{Pb}} = 495.08, \mathrm{pm}$), we evaluate the lattice mismatch between Pb and Cu, as shown in Equation 36.[S3,S4]

$$f_{\mathrm{Pb}\rightarrow\mathrm{Cu}} = \frac{a_{\mathrm{Pb}} - a_{\mathrm{Cu}}}{a_{\mathrm{Cu}}} \cdot 100\% = 36.94\% \tag{36}$$

Given this large mismatch, Pb adlayers on Cu are expected to be significantly strained and therefore cannot adopt the Cu lattice spacing. We thus use the Pb lattice parameter to define the unit area, while assuming that Pb atoms adopt the stacking registry imposed by the underlying Cu surface. The surface units derived from these assumptions, including the Pb overlayer used for the unit-charge construction on Cu(100), are shown in Figure S3.

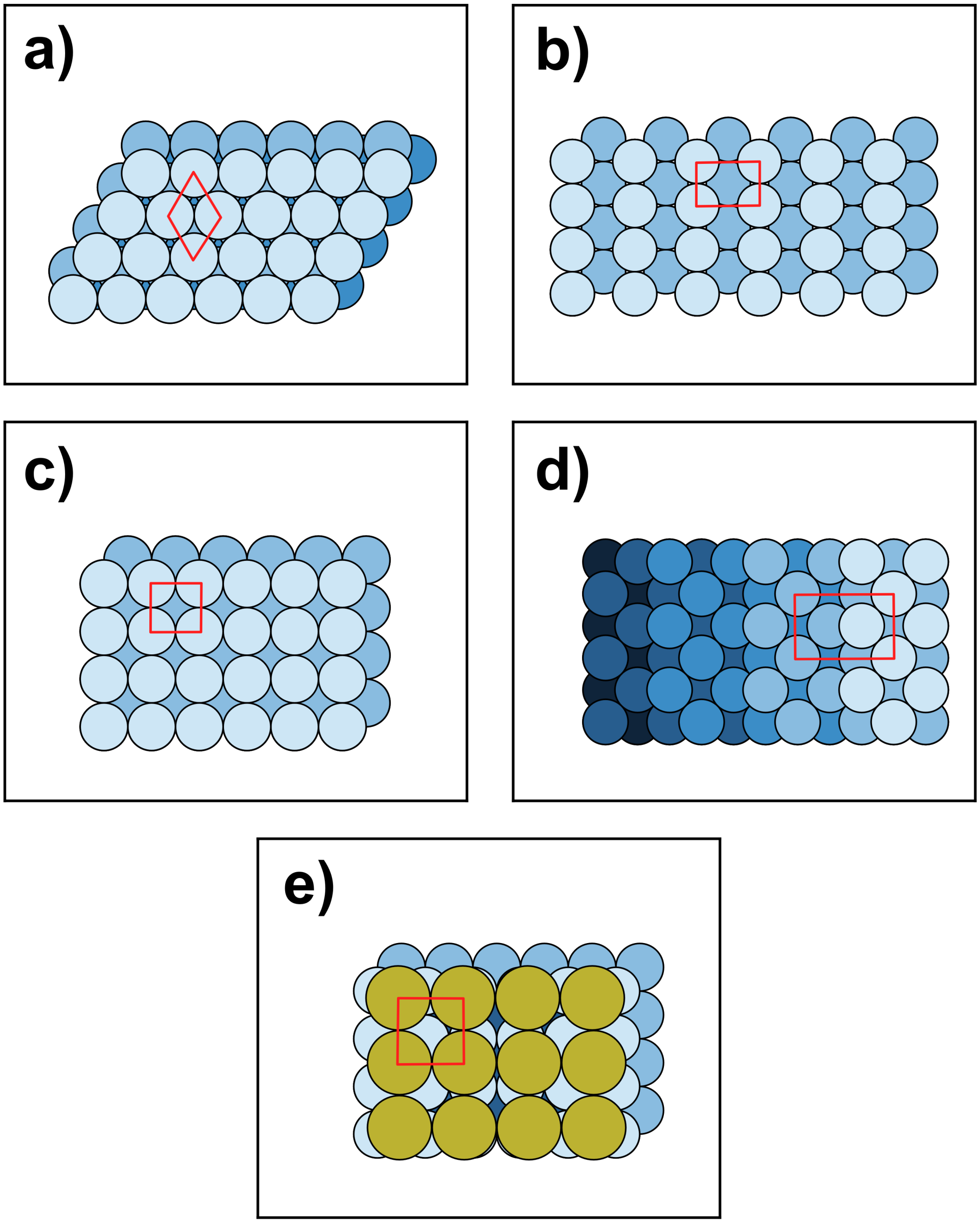

Figure S3: Representative periodic surface units for the crystallographic domains. Panels (a)–(d) show the Cu(111), Cu(110), Cu(100), and Cu(310) surfaces with their corresponding units marked in red. Panel (e) shows the assumed Pb overlayer structure on Cu(100) together with its periodic unit, used to define the unit charge for Pb-UPD.

5.2.1 Cu(111) Unit Charge

For Cu(111), the probe-relevant adsorption unit contains a single site (Equation 37).

$$N_{\text{sites,Cu(111)}} = 1 \tag{37}$$

The unit area constructed using the Pb lattice parameter is given in Equation 38.

$$A_{\text{unit,Cu(111)}} = \frac{\sqrt{3}\,a_{\text{Pb}}^2}{4} \tag{38}$$

The corresponding unit charge per unit area is shown in Equation 39.

$$\frac{Q_{\text{unit,Cu(111)}}}{A_{\text{unit,Cu(111)}}} = 302\,\mu\text{C}\,\text{cm}^{-2} \tag{39}$$

5.2.2 Cu(110) Unit Charge

For Cu(110), the probe-relevant adsorption unit contains a single site, as shown in Equation 40.

$$N_{\text{sites,Cu(110)}} = 1 \tag{40}$$

The unit area constructed using the Pb lattice parameter is given in Equation 41.

$$A_{\text{unit,Cu(110)}} = \frac{a_{\text{Pb}}^2}{\sqrt{2}} \tag{41}$$

The corresponding unit charge per unit surface area is shown in Equation 42.

$$\frac{Q_{\text{unit,Cu(110)}}}{A_{\text{unit,Cu(110)}}} = 185\,\mu\text{C}\,\text{cm}^{-2} \tag{42}$$

5.2.3 Cu(100) Unit Charge

For Cu(100), the probe-relevant adsorption unit contains a single site, as shown in Equation 43.

$$N_{\text{sites,Cu(100)}} = 1 \tag{43}$$

The unit area constructed using the Pb lattice parameter is given in Equation 44.

$$A_{\text{unit,Cu(100)}} = \frac{a_{\text{Pb}}^2}{2} \tag{44}$$

The resulting unit charge per unit surface area is shown in Equation 45.

$$\frac{Q_{\text{unit,Cu(100)}}}{A_{\text{unit,Cu(100)}}} = 261\,\mu\text{C}\,\text{cm}^{-2} \tag{45}$$

5.2.4 Cu(310) Unit Charge

For Cu(310), the probe-relevant adsorption unit contains two full sites, with an additional two-thirds site arising from the ABC stacking. The total number of sites is shown in Equation 46.

$$N_{\text{sites,Cu(310)}} = 2 + \frac{2}{3} \tag{46}$$

The unit area corresponds to the RSA of the Cu(310) surface unit. Because the vicinal geometry includes both terrace area and step-wall area, the step height ($h_{\text{step}} = a_{\text{Pb}}/2$) must be included when computing the RSA. The resulting unit area is given in Equation 47.

$$A_{\text{unit,Cu(310)}} = 2\,a_{\text{Pb}}^2 \tag{47}$$

The corresponding unit charge per unit surface area is shown in Equation 48.

$$\frac{Q_{\text{unit,Cu(310)}}}{A_{\text{unit,Cu(310)}}} = 174\,\mu\text{C}\,\text{cm}^{-2} \tag{48}$$

5.2.5 Computing the ECSA

Having established the unit charge per unit area for each crystallographic domain, we combine these quantities with the experimentally determined charge fractions to obtain the total ECSA. The corresponding expression is given in Equation 49.

$$A_{\text{ECSA}}^{(\text{sample})} = \sum_i x_{\text{Charge},i}\, Q^{(\text{sample})} \left(\frac{A_{\text{unit},i}}{Q_{\text{unit},i}}\right) \tag{49}$$

Substituting the domain-specific quantities for Cu(111), Cu(110), Cu(100), and Cu(310) yields the expression in Equation 50.

$$\begin{aligned} A_{\text{ECSA,Cu(poly)}}^{(\text{sample})} = x_{\text{Charge,Cu(111)}}\, Q_{\text{Cu(poly)}}^{(\text{sample})} \left(\frac{A_{\text{unit,Cu(111)}}}{Q_{\text{unit,Cu(111)}}}\right) \\ + x_{\text{Charge,Cu(110)}}\, Q_{\text{Cu(poly)}}^{(\text{sample})} \left(\frac{A_{\text{unit,Cu(110)}}}{Q_{\text{unit,Cu(110)}}}\right) \\ + x_{\text{Charge,Cu(100)}}\, Q_{\text{Cu(poly)}}^{(\text{sample})} \left(\frac{A_{\text{unit,Cu(100)}}}{Q_{\text{unit,Cu(100)}}}\right) \\ + x_{\text{Charge,Cu(310)}}\, Q_{\text{Cu(poly)}}^{(\text{sample})} \left(\frac{A_{\text{unit,Cu(310)}}}{Q_{\text{unit,Cu(310)}}}\right) \end{aligned} \tag{50}$$

Using the numerical values in Table S1, the surface-specific unit charges, and first-order error propagation, we obtain the result shown in Equation 51.

$$A_{\text{ECSA,Cu(poly)}} = 0.29 \pm 0.02\,\text{cm}^2 \tag{51}$$

The resulting ECSA is approximately 30% larger than the RSA. This would imply an effective probe coverage of 1.3, which is not physically meaningful for a metal underpotential deposition process. The most plausible explanation is uncertainty introduced by background subtraction, limitations in the experimental method, and the assumptions underlying the

unit-charge construction.

- **Background subtraction.** If the background was not fully removed from $Q_{\mathrm{Cu(poly)}}^{\mathrm{(sample)}}$, such that residual Q_{dl} or Q_{co} remained, the corrected faradaic charge would be overestimated and the resulting ECSA inflated.

- **Model limitation.** The linear-combination approach accounts only for ideal, well-defined crystallographic domains. Real polycrystalline Cu surfaces contain grain boundaries, step bunches, adatoms, vacancies, and kinks—none of which are represented in the model. If these features support a higher Pb-UPD charge per unit RSA than the ideal domains, the assumed full-coverage charge is underestimated and the calculated ECSA becomes artificially large.

- **Unit-charge assumption.** The unit charges were constructed using the Pb lattice parameter and an assumed one-to-one correspondence between Pb adsorption sites and the packing of the underlying Cu surface layer. If the Pb adlayer packs more densely, reconstructs, or experiences compressive strain, the true full-coverage charge would be higher, again leading to an overestimated ECSA.

We found a lattice mismatch between Pb and Cu of 37 %, which is comparable in magnitude to the approximately 30 % larger coverage implied by the ECSA estimate. Because Cu(110), Cu(100), and Cu(310) do not present close-packed surface arrangements, it is unlikely that a Pb overlayer can adopt an in-plane packing identical to these substrates. This suggests that our unit-charge assumptions may underestimate the true charge required for full Pb coverage on these orientations. This interpretation is consistent with prior observations in the literature.

Pb-UPD on Cu(111) is known to form an unrotated, incommensurate hexagonal overlayer whose nearest-neighbor spacing is compressed by 3.2 % relative to bulk Pb, as shown by in-situ surface XRD in 0.1 M $HClO_4$.[S5] In vacuum, Pb deposition on Cu(110) and

Cu(100) produces overlayers with superstructures that are denser than the substrate lattices, demonstrating that Pb can pack more closely than the underlying Cu surface.[S6,S7] These observations indicate that our unit-charge construction likely underestimates the true Pb site density at full coverage.

Viewed in this light, the ECSA example highlights both the strengths and the limitations of the approach. Performing the calculation yields atomistic insight into the Pb-UPD process and the structure of the Pb overlayer, but the unit-charge assumptions used here were not fully valid, and the resulting ECSA is therefore likely overestimated. More generally, determining an ECSA from an adsorption-limited probe requires an assumption about the maximum site density, and this assumption is difficult to justify when the adsorption geometry is not known. In such cases, uncertainties in the construction of unit charges per unit area propagate directly into the inferred ECSA.

5.3 Active Site Count Determination

For an adsorption-limited faradaic probe reaction such as Pb-UPD, the number of electrochemically addressable surface sites follows directly from the integrated charge and the electron stoichiometry of the probe reaction. The corresponding site count is given in Equation 52.

$$N_{\text{probe,active}}^{(\text{sample})} = \frac{Q_{\text{Cu(poly)}}^{(\text{sample})}}{n_{\text{probe}}\, e} \tag{52}$$

Using the background-corrected Pb-UPD charge and $n_{\text{probe}} = 2$, the number of electrochemically addressable Pb-UPD sites on the polycrystalline Cu is given in Equation 53.

$$N_{\text{probe,active}}^{(\text{sample})} = (2.3 \pm 0.2) \times 10^{14} \text{ sites} \tag{53}$$

5.4 Normalization to Electrocatalytic Current

Let us consider a target electrocatalytic reaction on the polycrystalline Cu electrode of the form shown in Equation 54.

$$\mathrm{Red} \longrightarrow \mathrm{Ox}^{+} + \mathrm{n}_{\mathrm{target}}\mathrm{e}^{-} \tag{54}$$

Suppose that a steady-state current of $I = 1\,\mathrm{mA}$ is measured at a fixed potential. Normalization by the RSA gives the current density in Equation 55.

$$j_{\mathrm{RSA}} = 4.5 \pm 0.4\ \mathrm{mA\,cm^{-2}} \tag{55}$$

If the Pb-UPD probe interrogates the same surface sites that participate in the target electrocatalytic reaction, the current may also be normalized by the ECSA and by the number of active sites.

Normalization by the ECSA is shown in Equation 56.

$$j_{\mathrm{ECSA}} = 3.9 \pm 0.3\ \mathrm{mA\,cm^{-2}} \tag{56}$$

Because the ECSA is likely overestimated, the corresponding current density appears artificially low. Normalization by the number of electrochemically addressable sites relies on fewer assumptions and provides a more direct physical measure. The site-normalized current is given in Equation 57.

$$I_{\mathrm{site}} = (3.4 \pm 0.3) \cdot 10^{-15}\ \mathrm{mA\,site^{-1}} \tag{57}$$

It can be more intuitive to express I_{site} in units of electrons transferred per second per site. Converting the site-normalized current to this representation gives Equation 58.

$$I_{\mathrm{site}} = 21 \pm 2\ \mathrm{e^{-}\,s^{-1}\,site^{-1}}. \tag{58}$$

The expression in Equation 58 is simply an alternative representation of I_{site}; the TOF definition below uses the electrical current form of I_{site} as given in Equation 57.

For a kinetically limited electrocatalytic process, the site-normalized current can be expressed as a turnover frequency (TOF), as shown in Equation 59.

$$\text{TOF} = \frac{I_{\text{site}}}{n_{\text{target}}\, e} \tag{59}$$

For a one-electron target reaction ($n_{\text{target}} = 1$), the turnover frequency becomes the value shown in Equation 60.

$$\text{TOF} = 27 \pm 2 \text{ s}^{-1} \tag{60}$$

Overall, the Pb-UPD example illustrates that ECSA values derived from adsorption-limited probes are sensitive to assumptions about the maximum achievable site density, which is rarely known for real electrocatalyst surfaces. When these assumptions are approximate, the ECSA can be systematically over- or underestimated. By contrast, the integrated charge provides a direct measure of the number of electrochemically addressable sites and therefore offers a more robust normalization metric. This metric is only meaningful, however, when the probe reaction samples the same surface sites that are active under the operational conditions of the target reaction, or at least provides a consistent proxy for their population.

References

(S1) Couce, P. M., Madsen, T. K., Plaza-Mayoral, E., Kristoffersen, H. H., Chorkendorff, I., Dalby, K. N., Van Der Stam, W., Rossmeisl, J., Escudero-Escribano, M., and Sebastián-Pascual, P. (2024) Tailoring the facet distribution on copper with chloride. *Chemical Science 15*, 1714–1725.

(S2) Taylor, J. R. *An Introduction to Error Analysis: The Study of Uncertainties in Physical Measurements*, 2nd ed.; University Science Books, 1997.

(S3) Klug, H. P. (1946) A.Redetermination of the Lattice Constant of Lead. *Journal of the American Chemical Society 68*, 1493–1494.

(S4) Straumanis, M. E., and Yu, L. S. (1969) Lattice parameters, densities, expansion coefficients and perfection of structure of Cu and of Cu–In phase. *Acta Crystallographica Section A 25*, 676–682.

(S5) Chu, Y. S., Robinson, I. K., and Gewirth, A. A. (1997) Properties of an electrochemically deposited Pb monolayer on Cu(111). *Physical Review B 55*, 7945–7954.

(S6) Nagl, C., Pinczolits, M., Schmid, M., Varga, P., and Robinson, I. K. (1995) $p(n \times 1)$ superstructures of Pb on Cu(110). *Physical Review B 52*, 16796–16802.

(S7) Nagl, C., Platzgummer, E., Haller, O., Schmid, M., and Varga, P. (1995) Surface alloying and superstructures of Pb on Cu(100). *Surface Science 331-333*, 831–837.
